# Plastid-nucleus communication involves calcium-modulated MAPK signalling

**DOI:** 10.1038/ncomms12173

**Published:** 2016-07-11

**Authors:** Hailong Guo, Peiqiang Feng, Wei Chi, Xuwu Sun, Xiumei Xu, Yuan Li, Dongtao Ren, Congming Lu, Jean David Rochaix, Dario Leister, Lixin Zhang

**Affiliations:** 1 Photosynthesis Research Center, Key Laboratory of Photobiology, Institute of Botany, Chinese Academy of Sciences, Beijing 100093, China; 2 University of Chinese Academy of Sciences, Beijing 100049, China; 3 State Key Laboratory of Plant Physiology and Biochemistry, College of Biological Sciences, China Agricultural University, Beijing 100094, China; 4 Departments of Molecular Biology and Plant Biology, University of Geneva, 1211 Geneva, Switzerland; 5 Division of Plant Molecular Biology (Botany), Department Biology I, Ludwig Maximilians University, 80638 Munich, Germany

## Abstract

Chloroplast retrograde signals play important roles in coordinating the plastid and nuclear gene expression and are critical for proper chloroplast biogenesis and for maintaining optimal chloroplast functions in response to environmental changes in plants. Until now, the signals and the mechanisms for retrograde signalling remain poorly understood. Here we identify factors that allow the nucleus to perceive stress conditions in the chloroplast and to respond accordingly by inducing or repressing specific nuclear genes encoding plastid proteins. We show that ABI4, which is known to repress the *LHCB* genes during retrograde signalling, is activated through phosphorylation by the MAP kinases MPK3/MPK6 and the activity of these kinases is regulated through 14-3-3ω-mediated Ca^2+^-dependent scaffolding depending on the chloroplast calcium sensor protein CAS. These findings uncover an additional mechanism in which chloroplast-modulated Ca^2+^ signalling controls the MAPK pathway for the activation of critical components of the retrograde signalling chain.

## Abstract (web summary)

Retrograde signalling co-ordinates nuclear gene expression in response to stress perceived in the chloroplast. Here Guo *et al.* show that a MAPK cascade acting downstream of a plastid derived Ca^2+^ signal contributes to the regulation of nuclear gene expression by phosphorylation of ABI4 during retrograde signalling.

## Graphical abstract

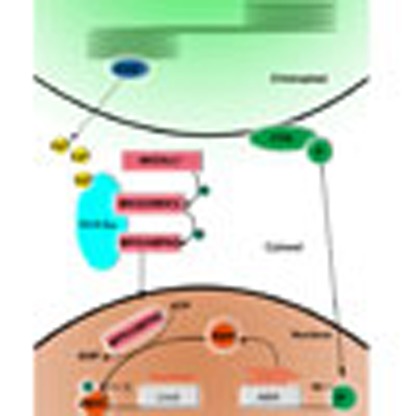

Chloroplasts are vital for the viability of plants. They are not only the site of photosynthesis, but also function as central hubs in plant metabolism^1^,^2^. Chloroplasts are considered semi-autonomous organelles in that they have their own genome although most chloroplast proteins are encoded by nuclear genes^3^. The functional properties of chloroplasts are tightly regulated by the nuclear genome. Intracellular chloroplast retrograde signalling regulates the nuclear gene expression and is essential for the biogenesis of chloroplasts and for maintaining optimal chloroplast function in response to fluxes of metabolites and changes in environmental conditions^4^,^5^. Therefore, in addition to their roles as sites of energy conversions and as metabolic hubs, chloroplasts also serve as environmental sensors and evoke a broad range of stress responses of plants^6^.

During the past two decades, genetic screening of *Arabidopsis* has identified six mutants with defects in retrograde signalling termed *genome uncoupled* (*gun*) mutants, in which the expression of photosynthesis-associated nuclear genes is partially derepressed on treatment with either norflurazon (NF), an inhibitor of carotenoid biosynthesis, or lincomycin (Lin), an inhibitor of plastid protein synthesis^4^,^5^. Studies of these mutants revealed the existence of multiple retrograde pathways. Perturbations of the tetrapyrrole pathway, changes in the reduction/oxidation state of the photosynthetic electron transfer chain (PET), inhibition of chloroplast gene expression (PGE) and production of reactive oxygen species (ROS) would trigger retrograde signalling^7^,^8^,^9^,^10^ and these signals were integrated by GUN1 within the chloroplast. In response to the GUN1-derived signal, the nuclear-localized AP2/ERF class transcription factor ABI4 represses photosynthesis-associated nuclear genes by competing with G-box binding factors (GBFs) for binding to the G-box^11^. In addition, metabolites such as the recently discovered haem, 3′-phosphoadenosine 5′-phosphate (PAP), β-cyclocitral and methylerythritolcyclodiphosphate (MEcPP) produced in stressed chloroplasts act as novel types of retrograde signals to regulate stress-responsive genes in the nucleus^12^,^13^,^14^,^15^,^16^. It is likely that multiple pathways and metabolites form a complex signalling network and are involved in transmitting the chloroplast signals to the nucleus to coordinate gene expression in response to environmental and developmental cues. However, how chloroplasts initiate the retrograde response and the nature of the retrograde signal moving out from the plastid to influence nuclear gene expression are not fully understood.

Perception and transduction of environmental and developmental cues within plant cells involve a set of interconnected intracellular signalling networks. Evolutionarily conserved three-tiered mitogen-activated protein kinase (MAPK) cascades are among the most thoroughly studied signalling cascades^17^. *Arabidopsis* MAPK cascades are activated by various endogenous and exogenous stimuli and participate in diverse cellular functions ranging from developmental processes such as stomata development, cell division and petal abscission to biotic and abiotic stress responses^18^,^19^,^20^. A key question in studies of this cascade is how the specificity of different cellular signals can be efficiently achieved through a common set of components. Scaffold proteins that bind several MAPK components contribute to the efficiency and specificity of signal transmission^21^. The role of scaffold proteins in the spatial-temporal organization and regulation of MAPK pathway is well established in higher eukaryotes. On the other hand, MAPK scaffold proteins could also mediate the crosstalk between signalling pathways. For example, the MAPK scaffold protein Ste5 in yeast links the MAPK cascade to G-protein signalling in the mating pathway through binding to G-protein βγ-subunits^22^. In mammals, the scaffold protein IQGAP1 couples Ca^2+^ signalling and MAPK signalling through Ca^2+^-mediated promotion of binding of IQGAP1 to B-Raf^23^.

Our previous studies showed that dysfunctional chloroplasts initiate the retrograde response by triggering the processing of PTM (PHD type transcription factor with transmembrane domains), a chloroplast envelope-bound plant homeodomain (PHD) transcription factor. The N-terminal PTM fragment is released and mediates activation of ABI4 at the transcriptional level which in turn leads to the repression of *LHCB*^24^. Transgenic plants overexpressing the ABI4 gene did not induce *LHCB* repression under normal growth conditions (Supplementary Fig. 1), implying that the presence of ABI4 is necessary, but not sufficient for *LHCB* repression. In this study, we reveal another regulatory module in retrograde signalling that involves phosphorylation-dependent activation of ABI4 through the MPK3/MPK6 cascade. Activation of MAPK involves generation of a cytosolic Ca^2+^ transient mediated by the chloroplast Ca^2+^-binding protein CAS. In addition, we show that calcium-binding protein 14-3-3ω links chloroplast-modulated Ca^2+^signalling to MAPK signalling during retrograde response through its Ca^2+^-dependent scaffolding function.

## Results

### MPK3/MPK6 are ABI4-interacting proteins

**Figure 1:**
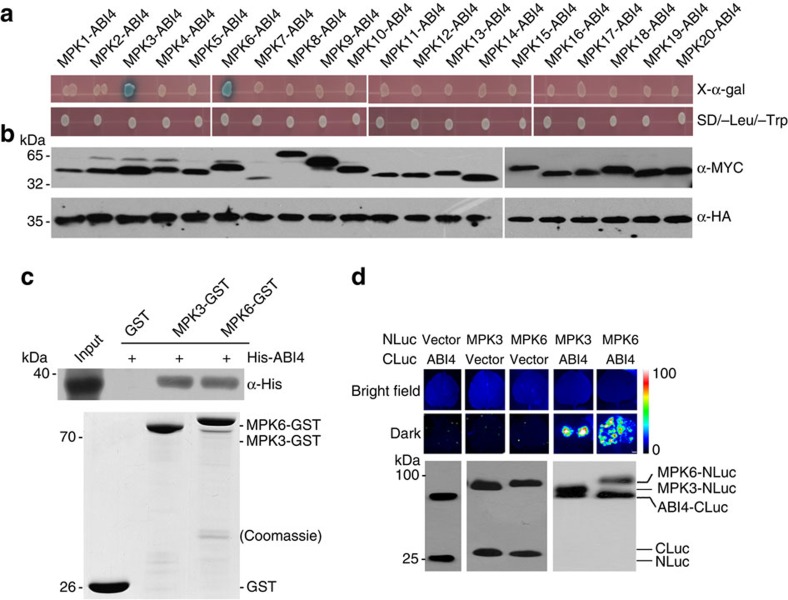
ABI4 interacts with MPK3 and MPK6. (**a**) Yeast two-hybrid assay for the interaction between ABI4 and MPK3/MPK6. Fusion constructs of the ABI4 (amino acids 1–160) fused with the GAL4 DNA-binding domain (BD) and 20 MAPKs fused with the GAL4 activation domain (AD) were co-transformed into Y2H Gold yeast cells. These strains were grown on SD-Leu-Trp medium (bottom) and subjected to α-galactosidase assay (top). (**b**) Immunoblot analysis of target fusion proteins in the yeast two-hybrid assays indicated in (**a**) using HA tag and Myc tag antibodies, respectively. (**c**) Pull-down assay for the interaction of ABI4 with MPK3/MPK6. His-tagged ABI4 was incubated with immobilized GST or GST-tagged MPK3/MPK6. After washing, bound proteins were eluted and subjected to immunoblot analysis using an antibody against His. Coomassie blue (CBB) staining indicates equal amounts of bait proteins. (**d**) Luciferase complementation imaging assay for the interaction of ABI4 with MPK3/MPK6. *N. benthamiana* leaves were co-infiltrated with the agrobacterial strains harbouring the construct pairs of ABI4-CLuc and MPK3/MPK6-NLuc. ABI4-CLuc and NLuc as well MPK3/MPK6-NLuc and CLuc were used as controls. The images were taken after 48 h of infiltration. Bright luminescence indicates direct protein–protein interactions and the pseudocolour bar shows the range of luminescence intensity in the images. The western blot below shows the expression levels of CLuc- and NLuc-fusion proteins using anti-full-length firefly LUC antibody. Scale bar, 1 cm.

The observations that *LHCB* gene expression was not suppressed in *ABI4* overexpressing transgenic plants under normal growth conditions in the absence of norflurazon (NF) despite the accumulation of ABI4 protein (Supplementary Fig. 1) suggests that a further activation step, possibly at the post-transcriptional level, is required for the ABI4-mediated retrograde response. Thus, the activity of ABI4 may be regulated either by post-translational modifications or through direct protein interactions. To address this possibility, we performed yeast two-hybrid screens using ABI4 lacking its activation domain to isolate interaction partners. Among these we isolated three clones encoding MPK6 (Supplementary Table 1). Because the *Arabidopsis* genome contains 20 MPK genes which belong to four subfamilies^25^, we subsequently tested whether other members of the MPK family are also able to directly interact with ABI4 in the yeast two-hybrid system. As shown in Fig. 1a, in addition to MPK6, MPK3, which is often functionally redundant to MPK6, interacted with ABI4 in the yeast two-hybrid system. The levels of the proteins expressed in yeast were checked by immunoblot analysis (Fig. 1b).

To provide further evidence for such interactions, we performed a pull-down assay. On immobilization of recombinant GST-MPK3/MPK6 fusion proteins on glutathione Sepharose beads, we found that both GST-MPK3 and GST-MPK6, but not GST alone, were able to pull down ABI4 *in vitro* (Fig. 1c). Furthermore, co-expression of ABI4 and MPK3 (or MPK6) in tobacco leaves resulted in strong LUC (firefly luciferase) activity, as demonstrated by luciferase complementation imaging (Fig. 1d). In control experiments when each of the three proteins alone was expressed LUC activity reached only background levels. Taken together, these results confirmed that ABI4 could interact with both MPK3 and MPK6 *in vitro* and *in vivo*.

### Phosphorylation of ABI4 by MPK3 and MPK6

**Figure 2:**
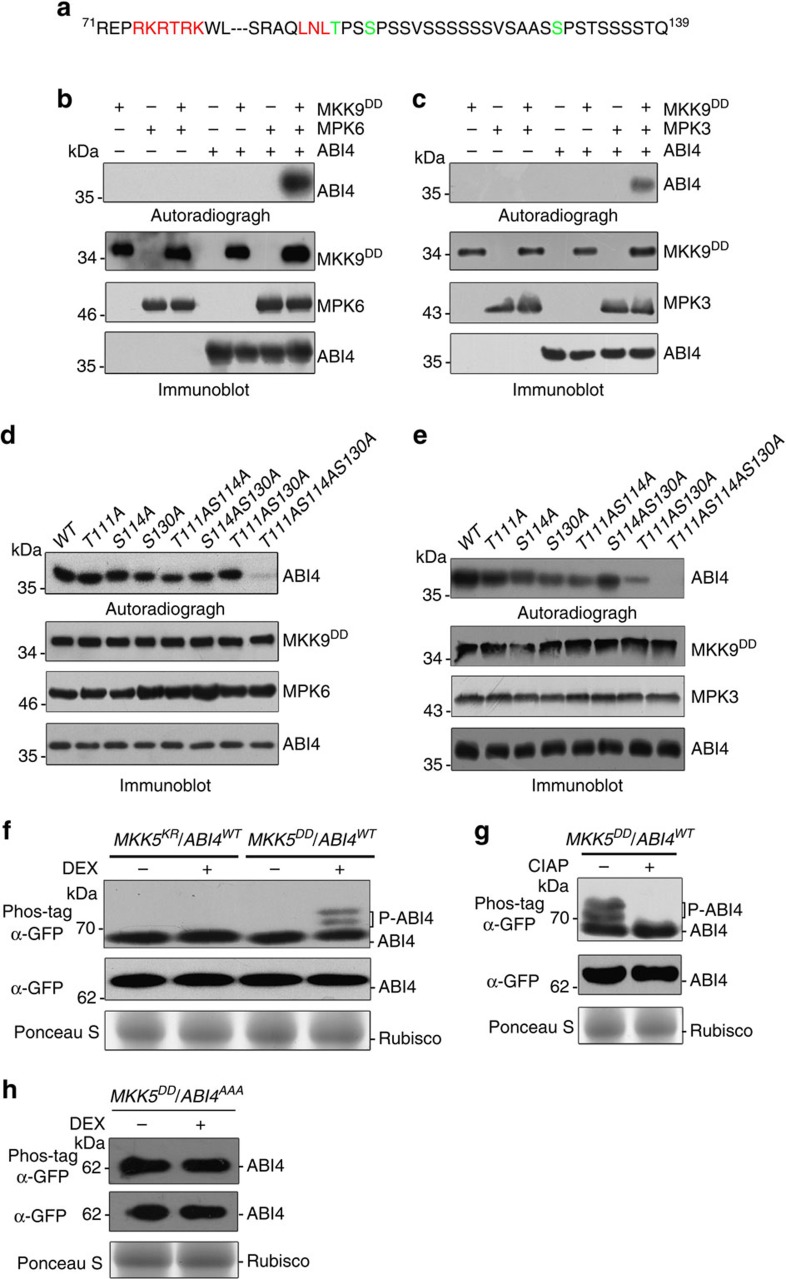
Phosphorylation of ABI4 by MPK3 and MPK6. (**a**) Putative MAPK docking motif and three potential phosphorylation sites in the N terminus of ABI4. The MAPK docking motif is highlighted in red, and the potential phosphorylation sites are highlighted in green. (**b**,**c**) Phosphorylation of recombinant ABI4 by the activated MPK3 and MPK6 *in vitro*. Recombinant MPK3/MPK6 were first activated by MKK9^DD^ and subsequently incubated with ABI4 in the presence of [γ-^32^P]ATP. Autoradiography was used to visualize phosphorylated proteins (upper panel) and equal input of MKK9^DD^, MPK3/MPK6 and was confirmed by immunoblot analysis (lower panel). Reactions with various components omitted (−) were used as controls. (**d**,**e**) Identification of the MPK3/MPK6 phosphorylation sites in ABI4 based on *in vitro* phosphorylation assays. Recombinant wild type and mutant ABI4 proteins with single, double and triple mutations were subjected to phosphorylation assays with preactivated recombinant MPK3/MPK6 in the presence of [γ-^32^P]ATP. The phosphorylation level was visualized by autoradiography (upper panel) and the loading of MKK9^DD^, MPK3/MPK6 and ABI4 proteins were determined by immunoblot analysis. (**f**) Phosphorylation of ABI4 is induced in *MKK5*^*DD*^*/ABI4*^*WT*^ but not in *MKK5*^*KR*^*/ABI4*^*WT*^ transgenic plants after DEX treatment. Total protein was prepared at 3 h after DEX treatment, separated in a Phos-tag SDS–PAGE and immunodetected by an anti-GFP antibody (top). A second immunoblot was performed to detect ABI4 (middle) and Ponceau S staining was used to demonstrate equal loading (bottom). (**g**) Characterization of up-shifted band of ABI4 by dephosphorylation. The protein extracts from DEX-treated *MKK5*^*DD*^*/ABI4*^*WT*^ seedlings were incubated with or without calf intestinal alkaline phosphatase (CIAP) and then subjected to Phos-tag SDS–PAGE (top) and SDS–PAGE analyses (middle). The ABI4^WT^-GFP protein was detected by immunoblot analysis using anti-GFP antibody. Ponceau S staining was used to demonstrate equal loading (bottom). (**h**) Effect of mutation of MAPK-phosphorylation sites on ABI4 phosphorylation status in the *MKK5*^*DD*^ background after DEX treatment. Protein extracts from *MKK5*^*DD*^*/ABI4*^*AAA*^ were prepared at 3 h after DEX treatment, separated in a Phos-tag SDS–PAGE and immunodetected by an anti-GFP antibody (top). Another immunoblot analysis was done at the same time to detect the ABI4 protein (middle). Ponceau S staining was used to demonstrate equal loading (bottom).

The direct interaction of ABI4 with MPK3 and MPK6 led us to examine whether ABI4 might also be phosphorylated by MPK3 and MPK6. The ability of MAPKs to phosphorylate their substrates is mediated by docking sequences and phosphorylation sites. The docking sequence is a common determinant of both the specificity and efficiency by which substrate proteins are phosphorylated by MAP kinases^26^. Its characteristic features include a cluster of basic residues located upstream of the LxL motif and sometimes a triplet of hydrophobic residues. The phosphorylation sites of substrates by MPKs are serine or threonine followed by proline (S/T-P motif)^27^. Sequence analysis indicated that the N terminus of ABI4 contains a typical MAPK docking motif and three potential phosphorylation sites (Thr-111, Ser-114 and Ser-130, designated as T111, S114 and S130, respectively) (Fig. 2a). *In vitro* phosphorylation assays showed that ABI4 was strongly phosphorylated in the presence of activated recombinant MPK3 and MPK6 (Fig. 2b,c). To identify which amino-acid residues are responsible for phosphorylation, recombinant ABI4 proteins were mutated by replacing Ser (S) or Thr (T) residues with Ala (A) and used as substrates for *in vitro* MAPK phosphorylation assays. Changes of all the three corresponding residues to Ala (ABI4^AAA^) in ABI4 led to a loss of phosphorylation whereas MPK3/MPK6 was still able to phosphorylate various single and double mutants (Fig. 2d,e). These results indicate that ABI4 is a substrate of MPK3/MPK6 and that the T111, S114 and S130 are independent MPK3/MPK6 phosphorylation sites.

To further test whether phosphorylation of ABI4 is dependent on MPK3 and MPK6 activities *in vivo*, we expressed a ABI4–GFP fusion protein in the conditional gain-of-function *MKK5*^*DD*^and loss-of-function *MKK5*^*KR*^ transgenic plants^28^, in which activation of MPK3/MPK6 is constitutively induced and abolished following DEX treatment, respectively (Supplementary Fig. 2). Phosphorylation of ABI4 was subsequently investigated by Phos-tag gel blot analysis with an anti-GFP antibody. In the gel containing Phos-tag, the phosphorylated proteins specifically binds to the Phos-tag reagent and cause a mobility shift compared with that of the non-phosphorylated counterparts^29^. As shown in Fig. 2f, phosphorylation of ABI4 was induced in the *MKK5*^*DD*^ background, but not in *MKK5*^*KR*^ plants, after DEX treatment, as indicated by the upshift of the protein bands. The band shifts of ABI4 were abolished after treatment with calf intestine alkaline phosphatase (CIAP), demonstrating that the additional bands with reduced mobility indeed result from phosphorylation (Fig. 2g). Such upshift was also absent when changes of all the three corresponding residues to Ala (ABI4^AAA^) in ABI4 under the *MKK5*^*DD*^ background after DEX treatment (Fig. 2h), again indicating that the identified MAPK phosphorylation sites were subject to phosphorylation. Together, these results indicate that ABI4 could indeed be phosphorylated on MPK3/MPK6 activation both *in vitro* and *in vivo*.

### Role of ABI4 phosphorylation during the retrograde response

**Figure 3:**
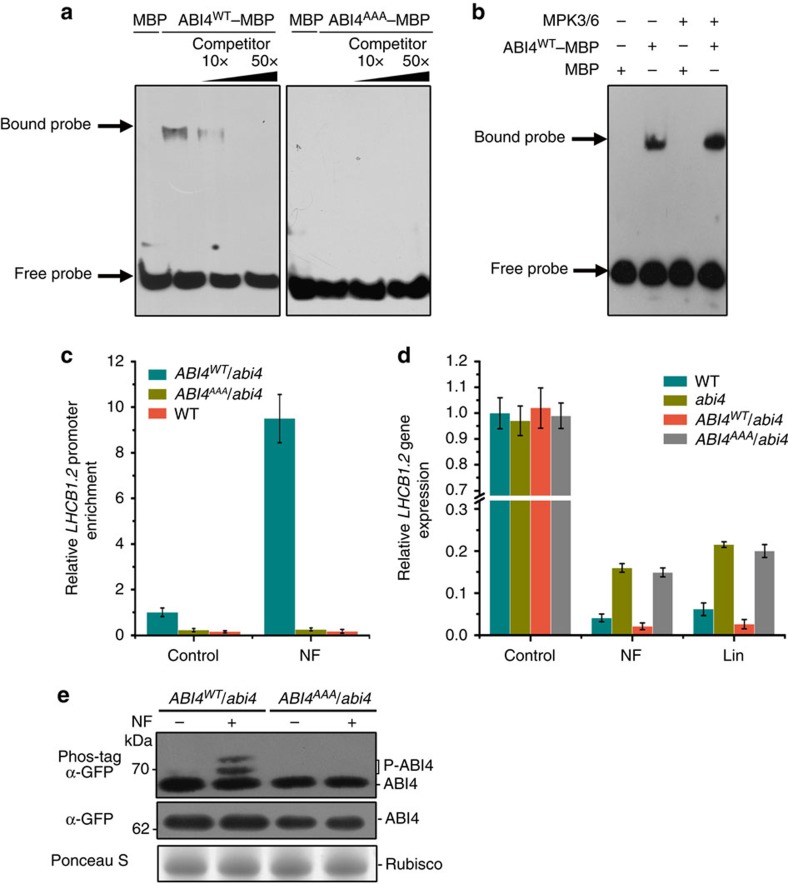
Phosphorylation of ABI4 is required for its function during the retrograde response. (**a**) The effect of mutations of phosphorylation sites on ABI4 DNA-binding activity by EMSA analysis. Recombinant ABI4^WT^ and ABI4^AAA^ proteins were incubated with a biotin-labelled *LHCB* promoter probe and detected by chemiluminescence. Increasing amounts of unlabelled competitors were used for competition and maltose-binding protein (MBP) served as a negative control. (**b**) ABI4 binding activity to the *LHCB* promoter upon MAPK-mediated phosphorylation. After incubation with activated MPK3 and MPK6 (equal mixture), phosphorylated ABI4 was subject to EMSA. MBP served as a negative control. (**c**) ChIP-qPCR analysis of ABI4 binding to the *LHCB* promoter in *ABI4*^*WT*^*/abi4* and *ABI4*^*AAA*^*/abi4* transgenic plants. Chromatin was isolated from 7-day-old seedlings in the presence or absence NF treatment. The GFP-tagged ABI4–chromatin complex was immunoprecipated using anti-GFP antibody. The relative DNA enrichment was determined by real-time PCR using primers specific to the promoters of *LHCB1.2*. A control reaction was processed using wild-type *Arabidopsis* samples. Values shown are means±s.d. of three biological replicates. (**d**) Impact of lincomycin (Lin) and NF treatments on *LHCB1.2* transcript levels in WT, *abi4*, *ABI4*^*WT*^*/abi4 a*nd *ABI4*^*AAA*^*/abi4.* Seven-day-old seedlings of WT, *abi4*, *ABI4*^*WT*^*/abi4 a*nd *ABI4*^*AAA*^*/abi4* grown on 1/2 MS media containing 500 μM Lin or 5 μM NF were harvested and RNA was isolated. The *LHCB* mRNA levels were determined by qRT–PCR. The relative transcript level of *LHCB* in seedlings grown on Lin or NF medium were normalized to values of control seedlings grown on 1/2 MS medium. Values shown are means±s.d. of three biological replicates. (**e**) Phosphorylation of ABI4 in seedlings after NF treatment. Total protein was extracted from *ABI4*^*WT*^*/abi4* and *ABI4*^*AAA*^*/abi4* seedlings at 0.5 h after NF treatment and separated in a Phos-tag SDS–PAGE followed by immunoblot analysis with anti-GFP antibody (top). Total protein extracts were separated by SDS–PAGE and the ABI4 protein was immunodetected with anti-GFP antibody at the same time (middle). Ponceau S staining was used to demonstrate equal loading (bottom).

The three phosphorylation sites of ABI4 lie in its DNA-binding domain, which raised the possibility that phosphorylation affects its DNA-binding activity. Therefore, we examined the effect of mutations of the phosphorylation sites of ABI4 on its DNA-binding activity using an electrophoresis mobility shift assay (EMSA). As shown in Fig. 3a, a protein–DNA complex was detected when recombinant ABI4 was incubated with the *LHCB* promoter. Competition experiments using an unlabelled probe of the *LHCB* promoter confirmed binding specificity. However, compared with wild type, mutant ABI4 lacking the phosphorylation sites lost DNA-binding activity, suggesting that MPK3/MPK6 phosphorylation sites are required for the DNA-binding activity of ABI4. In addition, the DNA-binding activity of ABI4 was further increased when ABI4 was phosphorylated by activated MPK3/MPK6 (Fig. 3b), demonstrating that the DNA-binding activity of ABI4 is promoted by MAPK-mediated phosphorylation. The result also implied that phosphorylated ABI4 may increase its binding capacity to its target genes *in vivo*. To test this, we performed a chromatin immunoprecipitation (ChIP) assay with an anti-GFP antibody on the *LHCB* promoter in the GFP-tagged ABI4^WT^ and ABI4^AAA^ transgenic plants and revealed that the changes of the phosphorylation sites to Ala residues significantly decrease the DNA-binding activity of ABI4 *in vivo* (Fig. 3c). Moreover, the enrichment of the *LHCB* promoter precipitated from the *ABI4*^*WT*^*/abi4* lines, but not from the *ABI4*^*AAA*^*/abi4* lines, was considerably increased after NF treatment. Together, these results demonstrated that MAPK phosphorylation sites are important for the DNA-binding activity of ABI4 and that MPK3/MPK6-mediated phosphorylation could enhance its DNA-binding capacity.

We next investigated the significance of ABI4 phosphorylation in chloroplast retrograde signalling. Analysis of *LHCB* mRNA level in *ABI4*^*WT*^*/abi4* revealed that the *gun* phenotype of *abi4* can be rescued by constitutive expression of ABI4^WT^ but not by ABI4^AAA^ protein lacking the MPK phosphorylation sites, suggesting that the phosphorylation of ABI4 is required for its action in retrograde signalling (Fig. 3d). Moreover, an *in vivo* phosphorylation assay showed that NF treatment induces the accumulation of phosphorylated ABI4 in *ABI4*^*WT*^*/abi4* but not in *ABI4*^*AAA*^*/abi4* plants (Fig. 3e). In agreement with the phosphorylation of ABI4, endogenous MPK6 and MPK3 activation could also be observed under NF or Lin treatment (Supplementary Fig. 3a,b). These results suggest that NF or Lin treatment induces the activation of MPK3/MPK6, which subsequently promotes ABI4 phosphorylation, and that the phosphorylation sites are required for the repression of *LHCB* expression during retrograde signalling.

### *ABI4* genetically interacts with *MPK3* and *MPK6*

**Figure 4:**
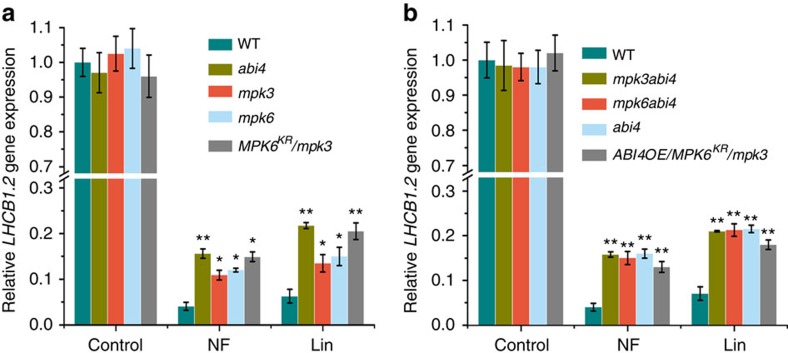
*ABI4* genetically interacts with *MPK3* and *MPK6* in the retrograde response. (**a**) Impact of Lin and NF treatments on *LHCB1.2* transcript levels in wild type(WT), *abi4*, *mpk3*, *mpk6* and *MPK6*^*KR*^*mpk3*. *Arabidopsis* seedlings were grown on 1/2 MS medium containing 500 μM Lin or 5 μM NF and harvested 7 days later. RNA was extracted and transcript levels of *LHCB* were analysed by qRT–PCR. (**b**) Impact of Lin and NF treatments on *LHCB1.2* transcript levels in WT, *abi4mpk3*, *abi4mpk6*, *abi4*, *ABI4OE* and *ABI4OE/MPK6*^*KR*^*/mpk3*. *Arabidopsis* seedlings were grown on 1/2 MS medium containing 500 μM Lin or 5 μM NF and harvested 7 days later. RNA was extracted and the levels of *LHCB* mRNA were analysed by qRT–PCR. The relative transcript level of *LHCB* in seedlings grown on Lin or NF medium were normalized to values of control seedlings grown on 1/2 MS medium. The mean ±s.d. of three biological replicates is shown. Asterisks indicate significant differences from WT (*P<0.05 or **P<0.01, Student's *t*-test).

Direct phosphorylation of ABI4 by MPK3/MPK6 suggests that MPK3/MPK6 may be involved in mediating a retrograde response. We next examined whether retrograde response is altered in the *mpk3* and *mpk6* mutants. As shown in Fig. 4a, after treatments with Lin and NF for 7 days, the transcript levels of *LHCB* decreased to almost 6 and 4% in wild-type seedlings, respectively. By contrast, ∼21 and 15% of *LHCB* transcript were retained in *abi4* after these treatments. As expected, Lin and NF treatments resulted in a reduction of *LHCB* to about 15% and 12% of the control in *mpk6* mutant, respectively. Similarly, the repression of *LHCB* transcripts by these inhibitors was less marked in *mpk3* mutants. Both *mpk3* and *mpk6* exhibit *gun* phenotypes under Lin and NF treatments, suggesting that MPK3 and MPK6 are indeed involved in the retrograde signal transduction pathway. Nevertheless, the *gun* phenotypes of *mpk3* and *mpk6* are weaker than that of *abi4*, which might be due to their functional redundancy. Because plants without both MPK3 and MPK6 display embryo lethality^30^, we employed *mpk3* lines expressing a dominant-negative MPK6 variant (*MPK6*^*KR*^) to assess the *gun* phenotype^31^. As expected, *MPK6*^*KR*^*mpk3* plants displayed a more severe *gun* phenotype that was similar to that of *abi4* (Fig. 4a).

To investigate the genetic interactions of *ABI4* with *MPK3* and *MPK6* in the retrograde response, we generated double mutants and analysis of double mutants revealed that *mpk3abi4* or *mpk6abi4* displayed a similar *gun* phenotype as the *abi4* single mutant (Fig. 4b), suggesting that ABI4 acts downstream of MPK3/MPK6. To further test whether MPK3 and MPK6 are required for *LHCB* transcript regulation, we overexpressed ABI4 in the *MPK6*^*KR*^*mpk3* mutant background. As shown in Fig. 4b, overexpression of ABI4 did not suppress the *gun* phenotype of *MPK6*^*KR*^*mpk3* plants, suggesting that MPK3/MPK6 regulates the activity of ABI4 at the post-transcriptional level rather than regulating its accumulation, which is in agreement with the role of MPK3/MPK6 in ABI4 phosphorylation. Collectively, these data suggest that ABI4 functions downstream of MPK3 and MPK6 in the retrograde pathway and ABI4-mediated suppression of *LHCB* transcription is largely dependent on full activation of MPK3/MPK6.

### MAPK activation involves CAS-mediated Ca^2+^ transients

**Figure 5:**
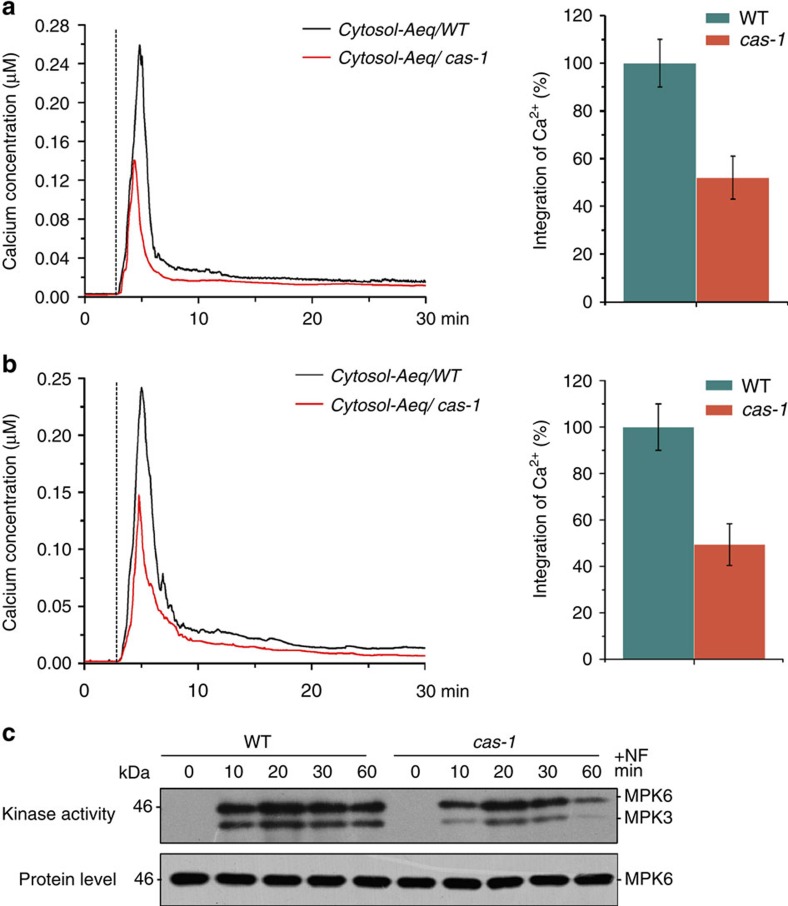
Activation of MAPK during retrograde response involves CAS-mediated transient increase in cytosolic Ca^2+^level. (**a**,**b**) Effect of NF and Lin on cytosolic calcium kinetics in transgenic *Arabidopsis* expressing apoaequorin in wild type (black lines) and *cas-1* mutant (red lines) plants. Six-day-old seedlings were treated with 5 μM NF (**a**) or 500 μM lincomycin (**b**) and luminescence was recorded at intervals of 0.2 s. Ca^2+^ concentrations were calculated from relative luminescence using a mathematical algorithm equation. The vertical dashed line indicates the time at which treatment was initiated. Experiments were repeated at least five times and representative data are shown. The time-integrated cytosolic Ca^2+^ concentration in wild-type and *cas-1* mutants in the first 30 min after treatment with 5 μM NF (**a**) or 500 μM lincomycin (**b**) is shown. Data are the means±s.d. of relative values of five replicates. At least five seedlings per genotype were measured in each replicate and wild-type values were set as 100%. (**c**) MPK3 and MPK6 activation profiles in WT and *cas-1* seedlings in response to NF treatment. Total protein was extracted after treatment with 5 μM NF for the indicated times and the activated MPK6 and MPK3 were detected by immunoblot analysis using phosphor-p44/42 MAPK (Erk1/2) antibody. Arrowheads indicate phosphorylated MPK6 and MPK3. Equal loading was indicated by immunoblot analysis with anti-MPK6 antibody.

The above results showed that MAP kinases mediate retrograde signalling by activating the downstream transcription factor ABI4. However, how the chloroplasts signal is emitted to regulate the activation of MAP kinases is unknown. Positive modulation of the Raf-MEK-ERK/MAPK cascade by an increase in intracellular Ca^2+^ is well established in animal cells^32^,^33^. Recent studies have demonstrated that chloroplasts serve as important intracellular calcium stores in plant cells, and they may also influence the entire cellular Ca^2+^ network by generating cytoplasmic Ca^2+^ transients^34^. To investigate a potential connection or overlap between MAPK and the Ca^2+^ pathway during the retrograde response, we first measured changes of calcium levels in the cytosol in response to retrograde signals using transgenic plants expressing the aequorin bioluminescence reporter^35^. As shown in Fig. 5a, challenging *Arabidopsis* seedlings with NF induced a rapid Ca^2+^ transient in the cytosol within several minutes, after which the signal gradually declined, implying that cytosolic calcium signals might play a role in meditating the chloroplast retrograde response. Because recent studies implied that the chloroplast calcium sensor protein CAS might be associated with cytosolic Ca^2+^ dynamics^35^,^36^, we measured NF-induced cytosol Ca^2+^elevation in aequorin-expressing plants in *cas-1* background. As shown in Fig. 5a, the amplitude of the signal was significantly attenuated in the *cas-1* mutant. The integrated amount of NF-induced cytosolic Ca^2+^ elevation over 30 min in *cas-1* mutant was reduced to about half of the wild-type amounts. Similar cytosolic Ca^2+^ transient patterns were observed after Lin treatment (Fig. 5b). Meanwhile, no obvious changes in cytosolic-free calcium were induced in control experiments in which water was employed instead of NF or Lin (Supplementary Fig. 4). Together, these results indicate that perturbation of plastid functions by NF and Lin causes a transient increase in cytosolic Ca^2+^ level in a CAS-dependent manner.

To investigate the possible role of chloroplast-modulated Ca^2+^ signalling in the regulation of MAPK signal pathway during retrograde signalling, we subsequently examined the effect of Ca^2+^ blockers on MPK3/MPK6 activity. We found that pretreatment with EGTA (a calcium-chelator) markedly suppressed Lin- and NF-triggered activation of MPK3 and MPK6. A similar but less pronounced effect was observed when applying lanthanum chloride, a calcium channel blocker (Supplementary Fig. 5a,b), suggesting that Ca^2+^ is positively involved in the modulation of MPK3/MPK6 activity during the retrograde response. Moreover, MAPK activation in response to retrograde signal was diminished in the *cas-1* mutant as compared with wild type (Fig. 5c), indicating that CAS significantly contributes not only to the cytosolic Ca^2+^ transient, but also to the downstream Ca^2+^-modulated cytosolic signalling response. NF is known to generate harmful ROS and cause photo-oxidative stress during illumination, which may lead to the activation of MPK3/MPK6 in *Arabidopsis*. Pretreatment with Tiron and dimethylthiourea (DMTU)^37^, scavengers for O_2_^−^ and H_2_O_2_, respectively, in dark-grown seedlings barely affected the activation of MPK3/MPK6 induced by NF treatment (Supplementary Fig. 5c), suggesting that ROS production does not play a major role in the activation of MPK3/MPK6.

### Calcium-binding protein 14-3-3ω facilitates MAPK activation

The observations that activation of MAPK in response to retrograde signals is regulated by chloroplast-modulated Ca^2+^ signalling prompted us to search potential signalling components that could link Ca^2+^ signals to the downstream MAPK cascades during the retrograde response. Scaffold proteins exert substantial control over MAPK signalling, ranging from signal intensity and specificity to crosstalk with other signalling cascades, and increasing evidence supports the existence of scaffold proteins in shaping a MAPK signalling module in *Arabidopsis*^38^,^39^. 14-3-3 proteins, a family of conserved scaffolding or anchoring proteins in eukaryotes for cellular signalling circuits, which can bind multiple signalling components, represent good candidates, as the association of 14-3-3s with some components of the MAPK cascade is well documented^40^,^41^,^42^.

**Figure 6:**
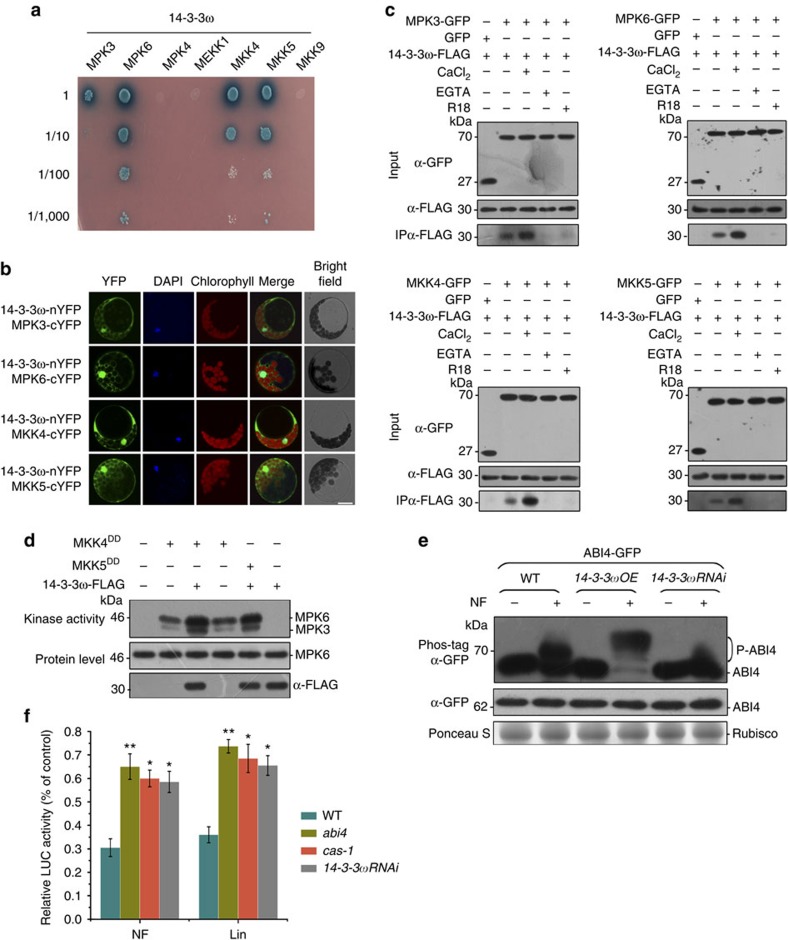
14-3-3ω facilitates MAPK activation through its Ca^2+^-dependent scaffolding role that is essential for the retrograde response. (**a**) Yeast two-hybrid assay for the interaction of 14-3-3ω with MPK3/MPK6 and MKK4/MKK5. These strains were grown on synthetic dropout medium lacking Leu, Trp and His (SD-Leu-Trp–His) containing 40 μg ml^−1^ X-α-gal (5-bromo-4-chloro-3-indolyl-α-D-galactopyranoside). (**b**) BiFC analyses for the interaction of 14-3-3ω with MPK3/MPK6 and MKK4/MKK5 in *Arabidopsis* protoplasts. YFP fluorescence was detected and the positions of nuclei were shown by 4, 6-diamidino-2-phenylindole (DAPI) staining. Scale bar, 10 μm. (**c**) Co-immunoprecipitation(co-IP) assays for the interaction of 14-3-3ω with MPK3/MPK6 and MKK4/MKK5. Protoplasts co-transfected with the MPK3/MPK6-GFP or MKK4/MKK5-GFP construct and the 14-3-3ω-FLAG construct were treated with 1 mM CaCl_2_, 5 mM EGTA or 10 μg ml^−1^ R18 for 1 h, and total protein extracts were subjected to immunoprecipitation. (**d**) Effect of 14-3-3ω on MAPK activation triggered by MKK4^DD^ or MKK5^DD^. Protoplasts were transfected with MKK4^DD^or MKK5^DD^ in the presence or absence of 14-3-3ω-FLAG constructs, and total protein extracts were subjected to immunoblot analysis with phosphor-p44/42 MAPK (Erk1/2) antibody. Equal loading was verified by immunoblot analysis with an anti-MPK6 antibody. (**e**) Phosphorylation status of ABI4 in wild-type, *14-3-3ωOE* and *14-3-3ωRNAi* plants expressing 35S:ABI4-GFP after NF treatment. Protein extracts from *ABI4OE*, *ABI4*^*W*T^*/14-3-3ωOE* and *ABI4*^*WT*^*/14-3-3ωRNAi* seedlings at 0.5 h after NF treatment were separated by Phos-tag SDS–PAGE followed by immunoblot analysis with anti-GFP antibody (top). Another immunoblot analysis was done at the same time to detect the ABI4 protein (middle). Ponceau S staining was used to demonstrate equal loading (bottom). (**f**) Effect of NF and Lin treatments on the relative activity of the LUC reporter in protoplasts. Protoplasts isolated from wild-type, *abi4*, *14-3-3ωRNAi* and *cas-1* mutant seedlings were cotransformed with both *LHCB*p:LUC and 35S:GUS constructs. Relative LUC activities after treatments are the ratio of LUC to GUS (internal control) normalized to the value of control (without treatment). Data represent means±s.d. from five biological replicates. Asterisks indicate significant differences from the value of wild-type at *P<0.05 or **P<0.01 using Student's *t* test.

We first investigated the expression profile of all 14-3-3 gene family members and found that 14-3-3ω exhibits a more rapid induction among the isoforms in response to retrograde signal (Supplementary Fig. 6), suggesting a possible role in the process. To test whether14-3-3ω exerts a regulatory role for MAPK pathway by acting as a scaffold during the retrograde signalling, we used the yeast two-hybrid method to test potential physical interactions between 14-3-3ω and components of the MAPK pathway. In *Arabidopsis*, MPK3 and MPK6 are activated probably through the well-studied MEKK1-MKK4/MKK5-MPK3/MPK6 kinase cascade^19^. As shown in Fig. 6a, 14-3-3ω specifically interacted with MKK4/MKK5 and MPK3/MPK6, but not with MEKK1. Also, MPK4, another well-characterized member of the MAPK family, as well as MKK9, another upstream MAPKK activator of MPK3 and MPK6, did not interact with 14-3-3ω in the yeast two-hybrid assays, demonstrating the specific interaction of 14-3-3ω with these MAPK pathway components. By contrast, the 14-3-3Φ isoform, the family member most closely related to 14-3-3ω (ref. 43), interacted with neither MPK3/MPK6 nor MKK4/MKK5 (Supplementary Fig. 7). Thus, we focused on 14-3-3ω in the subsequent studies. To confirm the association of 14-3-3ω with these MAPK components, we carried out transient bimolecular fluorescence complementation (BiFC) assays and found that coexpression of 14-3-3ω-nYFP (fusion of 14-3-3ω with the N-terminal fragment of yellow fluorescent protein (YFP)) with MPK3-cYFP (fusion of MPK3 with the C-terminal fragment of YFP), MPK6-cYFP, MKK4-cYFP or MKK5-cYFP produced strong YFP signals in both the nucleus and cytoplasm (Fig. 6b), consistent with the subcellular localization of these proteins^44^,^45^, while no fluorescence was detectable in the controls (Supplementary Fig. 8), indicating that MPK3, MPK6, MKK4 and MKK5 can interact with 14-3-3ω *in vivo*. Since it has been reported that 14-3-3ω undergoes conformational changes on binding of Ca^2+^at the C-terminal domain and since such binding of divalent cations is often required for the interaction of 14-3-3 with clients^46^,^47^, we next examined whether calcium could modulate the interaction between 14-3-3ω and MAPK cascade components *in vivo* using protoplast transient co-immunoprecipitated assays. Consistent with the yeast two-hybrid and BiFC assays, GFP-tagged MPK3, MPK6, MKK4 and MKK5, all co-immunoprecipitated with FLAG-tagged 14-3-3ω (Fig. 6c). The amounts of 14-3-3ω that were pulled down by MAPKK and MAPKs in the co-immunoprecipitation experiments significantly increased in the presence of Ca^2+^ (Fig. 6c). However, no 14-3-3ω could be immunoprecipitated when Ca^2+^ was chelated, suggesting that the 14-3-3ω and MPK3/MPK6–MKK4/MKK5 interaction was likely Ca^2+^ dependent (Fig. 6c). Likewise, treatment with R18 peptide, a strong competitive inhibitor of 14-3-3 client protein interactions^48^, also abolished such association (Fig. 6c). Moreover, a significant enhancement of the interaction between 14-3-3ω and the MKK4/MKK5–MPK3/MPK6 module could be observed following Lin and NF treatment in the wild type but such Lin- or NF-mediated promotion of interaction was dramatically reduced in *cas-1* mutants (Supplementary Fig. 9), which is consistent with the diminished MAPK activation in *cas-1* mutants. Together, these results indicate 14-3-3ω can interact with MKK4/MKK5–MPK3/MPK6 in a calcium-dependent manner and chloroplast-localized CAS enhances their association, possibly through modulation of cytoplasmic Ca^2+^ transients.

The association of 14-3-3ω with MKK4/MKK5 and MPK3/MPK6 suggests that it may function as a MAPK scaffold protein enabling spatial proximity. MAPK scaffold proteins would additionally increase the efficiency and specificity of MAPK activation by bringing MAPK cascade components into close proximity for physical assembly. We subsequently tested whether 14-3-3ω would have a similar stimulatory function in MAPK signalling in transfected protoplasts. As shown in Fig. 6d, expression of MKK4^DD^/MKK5^DD^, the constitutively active form of MKK4/MKK5 (ref. 49), can activate endogenous MPK3 and MPK6. As expected, the activation of MPK3 and MPK6 by MKK4^DD^ or MKK5^DD^ was significantly enhanced when the construct expressing epitope-tagged 14-3-3ω was cotransfected into protoplasts (Fig. 6d). By contrast, 14-3-3ω was unable to promote MAPK activation triggered by the constitutively active MKK9^DD^ (Supplementary Fig. 10), suggesting that such promotive effect of 14-3-3ω on MAPK activation was dependent on the interaction with its client protein. We next examined the effect of disrupting such 14-3-3 interaction via R18 treatment on MAPK activation and observed that the treatment with R18 peptide, but not with the inactive R18^Lys^ peptide, diminished the NF-mediated activation of MPK3 and MPK6 (Supplementary Fig. 11a). Similarly, the R18 peptide markedly suppressed the activation of MPK3 and MPK6 triggered by MKK4^DD^/MKK5^DD^, as well as the enhancing effect by14-3-3ω on MPK3 and MPK6 activation (Supplementary Fig. 11b), further supporting the notion that the enhancement of MAPK activation by 14-3-3ω was dependent on the interaction between 14-3-3ω and the MKK4/MKK5–MPK3/MPK6 module. Consistent with the protoplast transient assay, overexpression of 14-3-3ω significantly enhances MPK3/MPK6 activation, whereas downregulation of 14-3-3ω expression reduced MPK3/MPK6 activation as compared with wild-type plants on NF and Lin treatment (Supplementary Fig. 12). Accordingly, the NF-induced phosphorylation status of ABI4 was also increased by 14-3-3ω overexpression but attenuated in 14-3-3ω silencing background (Fig. 6e). These results collectively suggest that 14-3-3ω facilitates MAPK activation by functioning as a Ca^2+^-dependent scaffold for the MKK4/MKK5–MPK3/MPK6 module.

Finally, we examined the physiological significance of 14-3-3ω-mediated Ca^2+^-dependent scaffolding for MAPK signalling on the retrograde response. To this end, we monitored the activity of the *LHCB1.2* promoter (*LHCBp*) by transforming WT and *14-3-3ωRNAi* protoplasts with a *LHCBp:LUC* construct and measured LUC activity. In fact, NF or Lin treatments significantly suppressed LUC activity in WT but not in *14-3-3ωRNAi* protoplasts (Fig. 6f). Similarly, the extent of repression of LUC activity observed in the *cas-1* mutant or after pretreatment with Ca^2+^ blockers was much weaker(Supplementary Fig. 13), providing further evidence that CAS-generated cytosolic Ca^2+^ transients and Ca^2+^-dependent MAPK scaffolding function of 14-3-3ω are both required to repress the *LHCB* expression in response to retrograde signalling.

## Discussion

Coordinated communication between chloroplast and the nucleus are critical for proper chloroplast biogenesis and maintenance. Several components involved in the generation and transmission of retrograde signalling have been identified^4^,^5^. ABI4 has been proposed to competitively bind to promoter sequences and block the expression of photosynthetic genes in response to plastid signals^11^. Therefore, the involvement of ABI4 in plastid retrograde signalling is likely to be associated with a further activation step. So far, to our knowledge, no interacting partner has been identified to modulate the activity or stability of ABI4. In this study, we have elucidated the detailed mechanisms of ABI4 activation in retrograde signalling through MPK3/MPK6-mediated phosphorylation. Such phosphorylation-dependent activation is positively regulated by CAS-mediated cytosolic Ca^2+^ signalling and involves 14-3-3ω-mediated Ca^2+^-dependent scaffolding of the MAPK cascades.

*Arabidopsis* MAPK signalling pathways are involved in the regulation of various cellular processes. This includes responses related to biotic and abiotic stresses, hormone signalling and development programs^19^. Our results show that MPK3 and MPK6 play a prominent role during retrograde signalling by repressing the transcription of *LHCB* gene. Such multifunctionality of MPK3/MPK6 in different biological processes are considered to be conferred by their different substrates^50^. However, until now, only a few substrates have been reported with functional data^51^. Here we provided several lines of evidence supporting that ABI4 is a substrate of MPK3/MPK6. Both MPK3 and MPK6 interacted with ABI4 in yeast two-hybrid, pull-down and LIC assays. *In vitro* phosphorylation assays together with site-directed mutagenesis of the phosphorylated residues indicated that ABI4 can be phosphorylated by activated MPK3/MPK6. This suggestion was further corroborated by the Phos-tag mobility shift assays. Phosphorylation of the substrates of MPKs has been associated with changes in their stability and/or subcellular localization^19^,^52^. Recent studies have shown that ABI4 is regulated post-transcriptionally and protein stability is controlled through protein degradation involving the AP2-associated and the PEST motifs at the N-terminal region^53^,^54^. In our study, both the mutated ABI4^AAA^-GFP and wild-type ABI4^WT^-GFP were exclusively observed in the nucleus, suggesting that the subcellular localization of ABI4 was unaffected by phosphorylation (Supplementary Fig. 14). However, we observed that ABI4^AAA^-GFP is degraded much more rapidly compared with ABI4^WT^-GFP after addition of the protein synthesis inhibitor (Supplementary Fig. 15), implying that T111, S114 and S130 also contribute to the stability of ABI4. In our study, in the presence of NF or Lin, *LHCB* expression was much less repressed in *mpk3* and *mpk6* than in the wild type and more pronounced derepression of *LHCB* was observed in *MPK6*^*KR*^*mpk3* mutants. These results suggest that MPK3 and MPK6 play redundant roles, but both are required for mediating retrograde response. MPK3/MPK6 phosphorylation sites in ABI4 are within the DNA-binding domain. It has been proposed that a competition for the binding of the *LHCB* promoter between ABI4 and activators such as G-box binding factors (GBFs) occurs in the regulation of *LHCB* transcription^11^. Our EMSA and ChIP assay show that the binding activity of ABI4 to the *LHCB* promoter is positively regulated by phosphorylation. Further genetic analysis revealed that phosphorylation of ABI4 enhances its repression of *LHCB* transcription, because a non-phosphorylated mutant of ABI4 failed to rescue the *gun* phenotype of the *abi4* mutant. Based on these results, we conclude that ABI4 is activated by phosphorylation and that the phosphorylated active form of ABI4 predominantly binds the promoter of *LHCB* resulting in its repression during the retrograde response. A recent study has shown that a set of AP2/ERF transcriptional factors including ERF6, ERF104 and ERF105 exhibit rapid transcriptional upregulation after MPK6 activation downstream of the triose phosphate/phosphate translocator during high light-mediated retrograde signalling^55^. However, the transcription factors involved in these processes are unknown. Analyses of the promoters of these AP2/ERF genes revealed that the core element of the ABI4 binding motif (CCAC) was repeatedly present. The possibility of the involvement of phosphorylation of ABI4 in the transcriptional activation during this fast retrograde response to high light exposure can therefore not be excluded.

The involvement of Ca^2+^ in the retrograde response is supported by the finding that NF and Lin induce a rapid and transient Ca^2+^ increase in the cytosol, but this cytosolic Ca^2+^ transient was strongly diminished in *cas-1* mutants. These data suggest that perturbations of plastid tetrapyrrole biosynthesis or inhibition of plastid gene expression can elicit a transient increase in the cytosolic Ca^2+^ concentration. Such modulation of cellular Ca^2+^ signalling by chloroplasts during the retrograde response rely on chloroplast-localized CAS, possibly as a result of Ca^2+^ mobilization from chloroplasts in response to Lin and NF treatment. CAS-mediated Ca^2+^ signalling has previously been implicated in the retrograde control of immune responses and photoacclimation^56^,^57^. Although the results obtained in our study highlight that calcium released from chloroplasts contributes to MAPK activation during the retrograde response, the contribution of other internal Ca^2+^ stores for cytoslic Ca^2+^ transient cannot be excluded, because cytosolic Ca^2+^ transients were not completely abolished in *cas-1* mutants during the retrograde response. Meanwhile, the possibility that other tetrapyrrole intermediates mediate MAPK activation cannot be excluded. Impairment of chloroplast development might result in the generation of an inhibitory signal associated with the accumulation of certain chlorophyll intermediates. Thus, Mg-protoporphyrin IX (Mg-ProtoIX) has been reported to be involved in stress signalling and regulation of the activation of cyclin-dependent kinase A (CDKA) during the cell proliferation cycle in algae^58^,^59^.

Our study additionally suggests that a MAPK cascade links the CAS-mediated intracellular Ca^2+^ signalling to the nucleus to mediate the activation of ABI4 via the Ca^2+^-binding protein 14-3-3ω through its Ca^2+^-dependent scaffolding function.14-3-3 proteins have been implicated in the regulation of almost every plant cellular process through binding to a great variety of client proteins. The *Arabidopsis* genome contains at least 13 expressed 14-3-3 isoforms^57^. Our gene expression analysis showed that 14-3-3ω, but not other 14-3-3 family members, is dramatically induced by NF and Lin treatment, implying a distinct role for 14-3-3ω in the regulation of the retrograde response. We further demonstrated that calcium can promote the association between 14-3-3ω and the MKK4/MKK5–MPK3/MPK6 module. Because chelation of intracellular-free Ca^2+^ by EGTA abolishes such an interaction and the enhancement of the interaction in response to retrograde signalling was significantly reduced in the *cas-1* mutant, we postulate that 14-3-3ω acts as a Ca^2+^-dependent scaffold/anchor protein in MAPK signalling to facilitate MAPK activation by interacting with MKK4/MKK5 and MPK3/MPK6. Such an idea is further supported by the observation that 14-3-3ω overexpression results in the stimulation of MAPK signalling, whereas downregulation of 14-3-3ω by RNAi decreases MAPK activation. The findings collectively suggest that 14-3-3ω couples Ca^2+^ signalling to MAPK signalling during the retrograde response through Ca^2+^-dependent scaffolding, which may bring MPK3/MPK6 in close proximity to its activator kinase MKK4/MKK5, thereby enabling the efficient activation of MPK3/MPK6 and the subsequent phosphorylation of ABI4. Consistent with this notion, a transient expression assay revealed that *LHCB* repression upon Lin and NF treatment was less marked in CAS-deficient and 14-3-3ω-silenced background relative to wild type. Therefore, it seems that CAS-generated cytosolic Ca^2+^ transients and 14-3-3ω-mediated MAPK scaffolding both are essential events for the promotion of activation of ABI4 and subsequent suppression of *LHCB* during the retrograde signalling. In our study, because the MPK3/MPK6 do not contain canonical binding sites of the mode I [Arg-Ser-X-(pSer)-X-Pro] and mode II [Arg-X-X-X-(pSer)-X-Pro] for 14-3-3s interactions^60^, the interaction of 14-3-3ω with MPK3/MPK6 is likely mediated through non-canonical binding sites. The MKK4 amino-acid sequence of residues 2–8, RPIQSPP, and the MKK5 amino-acid sequence of residues 21–27, RPDLSLP, resemble the mode II recognition site. Replacing the Ser-6 of MKK4 and Ser-25 of MKK5 with Ala markedly reduced the interaction with 14-3-3ω (Supplementary Fig. 16), indicating that the Ser residue may serve as a 14-3-3ω recognition site in the yeast two-hybrid assay. However, these particular Ser residues have not been reported to be phosphorylated *in viv*o, so it cannot be excluded that Ser-6 of MKK4 and Ser-25 of MKK5 may have some structural function needed for signal transduction rather than act through their phosphorylation.

**Figure 7:**
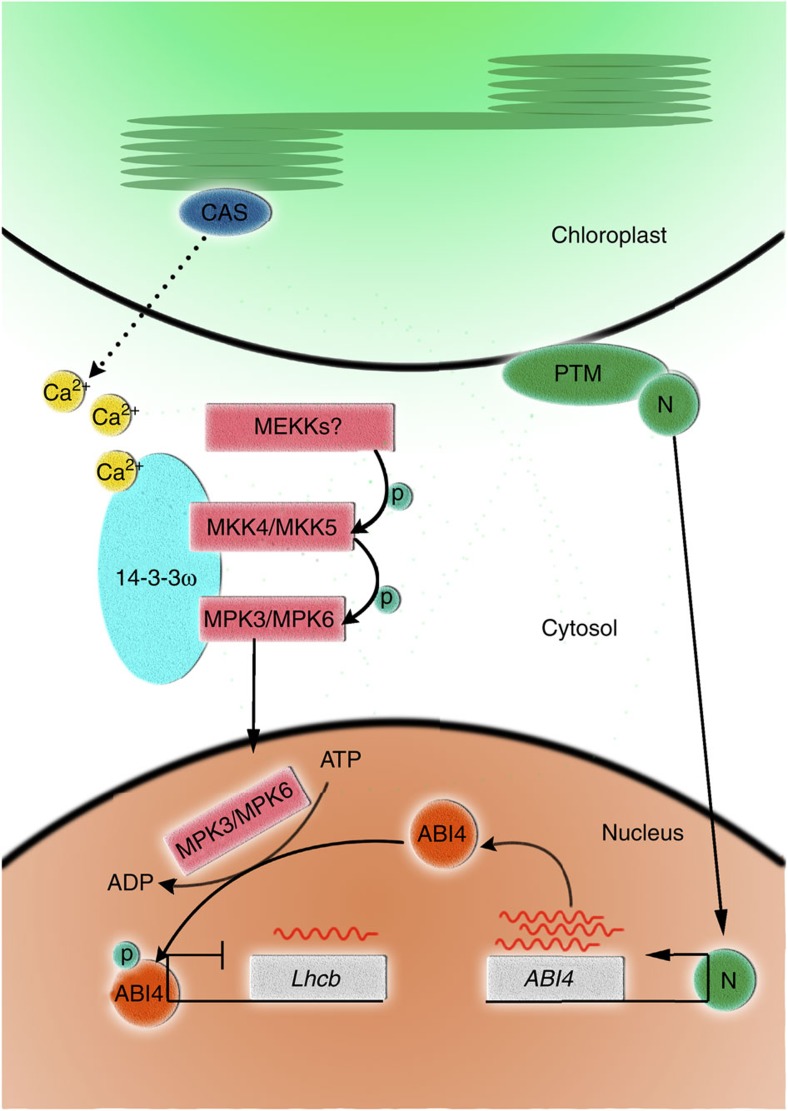
A model illustrating the transcriptional and posttranslational regulation of ABI4 by PTM and MPK3/MPK6 in retrograde signalling. Scheme illustrating the regulation of ABI4 activity during retrograde signalling by transcriptional (PTM) and post-translational (MPK3/MPK6) mechanisms. Perturbations of plastid tetrapyrrole biosynthesis or inhibition of plastid gene expression can elicit a transient increase in the cytosolic Ca^2+^ concentration, and thylakoid-localized CAS protein is involved in the generation of cytosolic calcium transients, possibly as a result of the Ca^2+^ mobilization from chloroplast in response to lincomycin and NF treatments. Subsequently, elevation of cytosolic calcium promotes the interaction between MKK4/MKK5–MPK3/MPK6 and the Ca^2+^-binding protein 14-3-3ω. 14-3-3ω functions as Ca^2+^-dependent scaffolding protein to bring MPK3/MPK6 in close proximity to its activator kinase MKK4/MKK5, thereby facilitating the efficient activation of MPK3/MPK6. Thus, relocation of activated MPK3/MPK6 into the nucleus together with phosphorylation of ABI4 is induced. In parallel, cleavage of PTM occurs and the released N-terminal fragment (designated as ‘N') moves to the nucleus where it activates the transcription of *ABI4*. Activation of ABI4 at both the transcriptional and posttranslational levels leads to the repression of *LHCB*.

In summary, we have identified several components of retrograde signal pathway involving ABI4 activation post-transcriptionally. These results, combined with our previous study on the role of PTM in the activation of ABI4 expression^24^, suggest that activation of ABI4 at both the transcriptional (by PTM) and post-translational levels (by MPK3/MPK6) leads to the repression of *LHCB* expression, as depicted in our working model (Fig. 7). Similar to PTM, disruption of MPK3/MPK6 also compromises the retrograde response. It is likely that both events are necessary for the repression of *LHCB* and that they act together in one regulatory pathway to coordinate photosynthesis-associated nuclear genes expression in response to retrograde signals.

## Methods

### Plant materials and growth conditions

The *abi4*, *mpk3* (Salk_100651), *mpk6* (Salk_127507), *cas-1*(Salk_070416) and *MKK5*^*DD*^ transgenic plants were described previously^24^,^28^,^35^. Homozygous mutant lines were identified by PCR analysis using specific primers (Supplementary Table 2). All the mutants used in this study are in the Columbia background. Wild type and mutant seeds were sterilized with 10% sodium hypochlorite for 10 min, washed five times with sterile water, and sown onto MS medium. To ensure synchronized germination, the seeds were kept in the dark at 4 °C for 2 days. All the plants were grown at 22 °C in a growth chamber under a 14 h light/10 h dark photoperiod at a photon flux density of 100 μmol photons m^−2^ s^−1^.

For NF treatment, the seeds were surface-sterilized and placed on half-strength MS media with or without the addition of 5 μM norflurazon (NF, Sandoz Pharmaceuticals). Plants were grown in the dark for 4 days and then transferred to light for 3 more days. For Lin treatment, the seeds were surface-sterilized and placed on half-strength MS media with or without the addition of 500 μM Lin (Sigma-Aldrich). After 7 days, the seedlings were harvested. For MAP kinase activity assays, 7-day-old seedlings were treated with 5 μM NF or 500 μM Lin for the indicated times. For DEX treatment, 7-day-old seedlings were collected for the indicated times following treatment with 2 mM DEX (Sigma-Aldrich). For LaCl_3_ and EGTA treatments, 7-day-old wild-type seedlings were treated with 2 mM LaCl_3_ (Sigma-Aldrich), 10 mM EGTA (Amresco) for 30 min prior to elicitation with NF or Lin treatment. For Tiron and DMTU treatments, the dark-grown 4-day-old wild-type seedlings were pretreated with 10 mM Tiron (Sigma-Aldrich) and 5 mM DMTU (Sigma-Aldrich) for 8 h, and then exposed to NF treatment in darkness for the time indicated.

### Generation of transgenic plants and double mutants

To construct the *ABI4*^*WT*^ and *ABI4*^*AAA*^ overexpression binary vector, the coding region of *ABI4* was amplified from wild-type complementary DNA using primers containing SalI and NcoI sites and inserted into the pGEM-T Easy vector (Promega) to produce pGEM-ABI4^WT^. To mutagenize the Thr-111, Ser-114 and Ser-130 residues to Ala, the fragments were amplified from pGEM-ABI4^WT^ template using the Fast Mutagenesis System Kit (TransGen Biotech) according to the manufacturer's protocol with primers listed in Supplementary Table 2. To generate ABI4^AAA^, all three Ser/Thr residues mutated to Ala by three successive mutagenesis steps to produce pGEM-ABI4^AAA^. After confirmation by sequencing, the wild-type and mutant fragments were released from pGEM-ABI4^WT^ and pGEM-ABI4^AAA^ with *Sal1*I and *Nco*I and ligated into the same restriction sites of the pCAMBIA2300 vector containing GFP as a marker under the control of the cauliflower mosaic virus 35 S promoter. The resulting constructs were electroporated into *Agrobacterium* strain C58C1 and then introduced into the WT or *abi4* background via the floral infiltration method^61^. Screening for kanamycin resistance was used to identify transformants. Independent lines were identified based on qPCR and immunoblot analysis with the anti-GFP antibody (G1544, Sigma-Aldrich) at a dilution of 1:4,000. The secondary antibody (goat anti-rabbit IgG, Sigma-Aldrich, A0545) conjugated to horseradish peroxidase (HRP) was used at a dilution of 1/10,000 for detection by the enhanced chemiluminescence assay.

For the generation of *MPK6*^*KR*^*mpk3* plants, the coding region of *MPK6* was amplified from wild-type cDNA using primers containing BamHI and XmaI sites and inserted into the pGEM-T Easy vector to produce pGEM-MPK6. To mutagenize the Lys to Arg mutation on the Lys-92 residue, the fragment was amplified from pGEM-MPK6 template using the Fast Mutagenesis System Kit according to the manufacturer's protocol with primers listed in Supplementary Table 2 to generate pGEM-MPK6^*KR*^. After confirmation by sequencing, the MPK6^*KR*^ fragments were released from pGEM-MPK6^*KR*^ with BamHI and XmaI and ligated together with a C-terminal FLAG epitope into the same restriction sites of pSN1301 binary vector to produce *35S:MPK6*^*KR*^. In addition, a 2,000-bp fragment upstream of ATG start codon of MPK6 was amplified from *Arabidopsis* genomic DNA, digested with HindIII and BamH1 and ligated into the same restriction sites of pSN1301 to replace the 35S promoter, resulting in *MPK6p: MPK6*^*KR*^. The resulting constructs were introduced into *Agrobacterium* strain C58C1 and transformed into *mpk3* background by floral infiltration. Screening for hygromycin resistance was used to identify transformants and expression of *MPK6*^*KR*^ proteins was identified based on qPCR and immunoblot analysis with the anti-FLAG M2 antibody(A8592, Sigma-Aldrich) covalently conjugated to HRPat a 1/4,000 dilution.

To generate *MKK5*^*KR*^ transgenic plants, the coding region of *MKK5* was amplified from wild-type cDNA using primers containing XhoI and SpeI sites and inserted into the pGEM-T Easy vector to produce pGEM-MKK5. To generate the Lys to Arg mutation on the Lys-99 residue, the fragment was amplified from pGEM-MKK5 template using the Fast Mutagenesis System Kit according to the manufacturer's protocol with primers listed in Supplementary Table 2, giving rise to pGEM-MKK5^*KR*^. After sequencing confirmation, the MKK5^*KR*^ fragments were released from pGEM-MKK5^*KR*^ with XhoI and SpeI and ligated into the same restriction sites of the steroid-inducible pTA7002 binary vector^28^. The resulting constructs were introduced into *Agrobacterium* strain C58C1 and transformed into wild-type *Arabidopsis* by floral infiltration. The transformants were identified by screening for hygromycin resistance.

To generate *MKK5*^*DD*^*/ABI4*^*W*T^ and *MKK5*^*KR*^*/ABI4*^*WT*^ transgenic plants, *MKK5*^*DD*^ and *MKK5*^*KR*^ were crossed into an *ABI4*^*WT*^ background, respectively. *MKK5*^*DD*^ and *MKK5*^*KR*^ were identified using hygromysin resistance, whereas the *ABI4*^*WT*^ were identified by kanamycin resistance and transgene expression. To generate *ABI4OE/MPK6*^*KR*^*/mpk3* transgenic plants, *MPK6*^*KR*^*/mpk3* was crossed into an *ABI4*^*WT*^ background. *MPK6*^*KR*^ was identified using hygromycin resistance, whereas *ABI4*^*WT*^ was identified by kanamycin resistance and transgene expression, and T-DNA insertions of MPK3 were identified by PCR of genomic DNA. Homozygous F3 plants from both crosses were used for the experiments. To generate *mpk3abi4* and *mpk6abi4* double mutants, the *mpk6* and *mpk3* mutants were crossed into *abi4* background. For genotyping of the progeny, the T-DNA insertions of MPK3 and MPK6 were identified by PCR of genomic DNA, whereas the *abi4* allele was identified on the basis of the ABA-insensitive phenotype. Homozygous F3 plants were used for the experiments.

To generate *14-3-3ω* overexpressing transgenic plants, the fragments encoding full-length of *14-3-3ω* fused with an N-terminal Flag epitope were amplified from wild-type cDNA, digested with XmaI and SacI and ligated into the same restriction sites of the pSN1301 binary vector under the control of a CaMV35S promoter. The resulting constructs were introduced into *Agrobacterium* strain C58C1 and transformed into wild-type *Arabidopsis* by floral infiltration. The transformants were identified by screening for hygromycin resistance and independent lines with the expression of FLAG-tagged 14-3-3ω were identified based on immunoblot analysis with the anti-FLAG M2 antibody (A8592, Sigma-Aldrich) covalently conjugated to HRP at a 1/4,000 dilution.

To generate *14-3-3ω* RNAi transgenic plants, a 423-bp cDNA fragment containing the last exon and 3′untranslated region of *14-3-3ω* was amplified by RT-PCR from total RNA extracted from wild-type seedling using primers containing NcoI and SwaI sites. After digesting with *Nco*I and *Swa*I, the resulting DNA fragment was ligated into the NcoI-SwaI sites pFGC5941 in sense orientation. Subsequently, the same fragment was amplified and inserted into downstream of chalcone synthase intron of pFGC5941 already containing the sense repeat using BamHI and SmaI sites. The resulting pFGC5941 vector contained the CaMV35S promoter-driven423-bp cDNA fragment of *14-3-3ω* in both sense and antisense orientations separated by a chalcone synthase intron. This resulting RNAi construct was electroporated into the *Agrobacterium tumefaciens* strain C58C1 and then introduced into the wild-type via the floral dip method. Transgenic plants were selected on MS plates in the presence of 50 μg ml^−1^ basta.

To generate *ABI4*^*W*T^*/14-3-3ωOE* and *ABI4*^*WT*^*/14-3-3ωRNAi* transgenic plants, *14-3-3ωOE* and *14-3-3ωRNAi* were crossed into an *ABI4*^*WT*^ background, respectively. *14-3-3ωOE* and *14-3-3ωRNAi* were identified using hygromycin and basta resistance, respectively, whereas the *ABI4*^*WT*^ were identified by kanamycin resistance. Homozygous F3 plants from both crosses were used for the experiments.

### RNA extraction and quantitative real-time PCR

Total RNA was extracted from seedlings by RNA extraction kit (Tiangen). After DNase I treatment, reverse transcription was conducted using PrimeScript II 1st Strand cDNA Synthesis Kit (Takara) according to the manufacturer's protocol. Real-time PCR was performed using an Mx3000p Real-time PCR System (Agilent, Stratagene) with the SYBR Premix ExTaq kit (Takara) in accordance with the manufacturer's instructions. For each sample, three replicates were performed and the expression levels were normalized to those of *UBQ*. All primers sequences were listed in Supplementary Table 2.

### Protein extraction and immunoblot analysis

Total protein was extracted from *Arabidopsis* using extraction buffer as described previously^62^. Briefly, plant material was ground in the Eppendorf tube using extraction buffer (125 mM Tris-HCl, pH 8.8, 1% (w/v) SDS, 10% (v/v) glycerol, 50 mM Na_2_S_2_O_5_), centrifuged at 13,000*g* for 10 min, and the supernatant was saved. Protein concentrations were determined using the Bio-Rad DC protein assay (Bio-Rad) following the manufacturer's instructions. For immunoblot analysis, total protein was separated by SDS–polyacrylamide gel electrophoresis (PAGE) and transferred to nitrocellulose membranes. The membranes were incubated with specific primary antibodies(GFP antibody, G1544, Sigma-Aldrich; FLAG M2 antibody, A8592, Sigma-Aldrich; MPK6 antibody, A7104, Sigma-Aldrich; Firefly Luciferase antibody, ab181640, Abcam). The secondary antibody (goat anti-rabbit IgG, Sigma-Aldrich, A0545) conjugated to HRP was used at a dilution of 1/10,000 for detection by the enhanced chemiluminescence assay. Uncropped images of blots/gels are shown in Supplementary Fig. 17.

### Assay of MAP kinase activity

For MAPK assay with seedlings, total protein was extracted from 7-day-old *Arabidopsis* seedlings using extraction buffer as described previously^63^. MAPK activation was monitored by immunoblot analysis using Phospho-p44/42 MAPK (Erk1/2) antibodies (#4370, Cell Signalling) at a dilution of 1/4000. The proteins were separated by 10% SDS–PAGE gels and immunodetected with antibody against MPK6 (A7104, Sigma-Aldrich) at a dilution of 1/4,000 to verify the same loading. The secondary antibody (goat anti-rabbit IgG, Sigma-Aldrich, A0545) conjugated to HRP was used at a dilution of 1/10,000 for detection by the enhanced chemiluminescence assay.

For MAPK assay in protoplasts, protoplasts were isolated from 4-week-old plants according to the protocol by Yoo *et al.*^64^. Briefly, 0.5–1 mm leaf strips were cut from fully expanded young leaves of 4-week-old *Arabidopsis* plants, and then transferred into the enzyme solution (20 mM MES, pH 5.7, 1.5% cellulase R10, 0.4% macerozyme R10, 0.4 M mannitol, 20 mM KCl, 10 mM CaCl_2_, 0.1% BSA) in the dark for at least 3 h at room temperature. After digestion, equal volume of W5 solution (2 mM MES, pH 5.7, 154 mM NaCl, 125 mM CaCl_2_, 5 mM KCl) was added and the enzyme solution was filtered using a 75-μm nylon mesh. The protoplasts were recovered through centrifugation at 100*g* for 1–2 min. The MKK5^DD^construct for protoplast expression was made by PCR amplifying the pET28-MKK5^DD^ plasmid^49^ and in-frame inserted between NcoI and XmaI sites of pSAT6-EYFP-N1, resulting pSAT6-MKK5^DD^, in which the stop codon was introduced at the N terminus of GFP. To generate MKK4^DD^ construct for protoplast assay, open reading frames of MKK4 were PCR amplified from *Arabidopsis* cDNA, cloned into pGEM-T vector (Promega), resulting in pGEM-MKK4. After confirmation by sequencing, the constitutively active form of MKK4 was generated by changing both putative phosphorylation sites to Asp residues (T224D and S230D) using the Fast Mutagenesis System Kit (TransGen Biotech) according to the manufacturer's protocol with primers listed in Supplementary Table 2, giving rise to pGEM-MKK4^DD^. After sequencing confirmation, the pGEM-MKK4^DD^ was digested with NcoI and XmaI and ligated into the same restriction sites of pSAT6-EYFP-N1, resulting pSAT6-MKK4^DD^, in which the stop codon was introduced at the N terminus of GFP. 14-3-3ω construct is the same as used in the CoIP assay. The combinations of the indicated plasmids were co-transformed into *Arabidopsis* protoplasts using PEG-calcium-mediated transformation. After overnight incubation at 23 °C, the protoplasts were lysed by vigorously vortexing for 1 min. The resultant clear lysate was centrifuged at 12,000*g* for 10 min at 4 °C, and the supernatant was separated on a 10% SDS–PAGE gel and subjected to immunoblot analysis using the phosphor-p44/42 MAPK (Erk1/2) antibody at a dilution of 1/4,000. The secondary antibody (goat anti-rabbit IgG, Sigma-Aldrich, A0545) conjugated to horseradish peroxidase was used at a dilution of 1/10,000 for detection by the enhanced chemiluminescence assay. For R18 peptide treatment, R18 peptide was added to the protoplasts at a final concentration of 10 μg ml^−1^ for 2 h prior to protein extraction.

### Measurements of Ca^2+^ concentrations

Stable transgenic *Arabidopsis* plants expressing cytosol-targeted apoaequorin under control of the 35S promoter with comparable amount of apoaequorin in wild-type and *cas-1* mutants background, respectively^35^, were used. Ca^2+^ concentration was determined using the method described by Knight *et al.*^65^. Briefly, 6-day-old seedlings were transferred from the Petri dish to an eppendorf tube containing 200 μl of reconstitution buffer (2 mM MES–NaOH, pH 5.7) containing 5 μM coelenterazine (Promega) and incubated overnight in the dark at room temperature. After overnight incubation, the aequorin luminescence was recorded every 0.2 s in GLOMAX20/20 Luminometer (Promega). After 3 min of counting for the base level, NF or Lin was injected into the tube at a final concentration of 5 μM and 500 μM, respectively. The luminescence was recorded immediately for the indicated time. At the end of the experiments, the remaining aequorin was discharged by the addition of an equal amount of discharging solution (2 M CaCl_2_ dissolved in 20% (v/v) ethanol). Calibration of aequorin luminescence was performed according to Alvarez *et al.*^66^

### Yeast two-hybrid assay

For yeast two-hybrid screening, experiments were performed using Matchmaker Gold Yeast Two-Hybrid System (Clontech) according to the manufacturer's instructions. Briefly, the bait, N-terminal fragments of ABI4 (amino acids 1–160), was cloned into *Nde*I and *Pst*I sites of pGBT7 vector (Clontech) and introduced into the yeast strain Y2H Gold (Clontech). The bait strain was grown in SD/-Trp liquid medium overnight and prepared for mating with library strain (Universal Normalized *Arabidopsis* cDNA library, Clontech) at 30 °C for 24 h. Then, yeast cells were pelleted, washed twice with 0.5 × YPDA and resuspended in 10 ml of 0.5 × YPDA. The resuspension culture was spread out on SD/-Leu/-Trp/-His plates in the presence of 125 ng ml^−1^ Aureobasidin A. After 4 days, the positive clones were picked up for a α-galactosidase activity assay and blue colonies were further used for plasmid extraction and sequencing.

To assay the interaction of ABI4 with the MAPKs, the N-terminal fragments of ABI4 (amino acids 1–160) and the coding regions of each member of *Arabidopsis* MAPKs were amplified by PCR with primers containing appropriate restriction sites and inserted into vectors of pGBK and pGAD, respectively. To assay the interaction of 14-3-3ω with the components of the MAPK pathway, fragments encoding full-length 14-3-3ω were amplified by PCR from wild-type cDNA and digested with EcoRI and PstI. The resulting fragments were inserted into the same restriction sites of pGBK plasmid. Full-length cDNAs corresponding to MKK4, MKK5, MKK9 and MEKK1 were amplified by PCR. The resulting fragments were digested by the appropriate restriction enzymes and subcloned in pGAD vector, to yield the vectors pGAD-MKK4, pGAD-MKK5, pGAD-MKK9, pGAD-MKK4, and pGAD-MEKK1. pGAD-MPK3, pGAD-MPK6 and pGAD-MPK4 constructs are the same as used in the ABI4–MAPKs interaction assay. To assay the interaction of 14-3-3Φ with the components of the MAPK pathway, full-length cDNA14-3-3Φ was amplified by PCR from wild-type cDNA and digested with NdeI and PstI. The resulting fragments were inserted into the same restriction sites of pGBK plasmid. PGAD-MPK3, pGAD-MPK6, pGAD-MKK4 and pGAD-MKK5 constructs are the same as used in the 14-3-3ω interaction assay. The MKK4^S6A^-pGAD and MKK5^S25A^-pGAD was generated from pGAD-MKK4 and pGAD-MKK5 vectors by changing indicated Ser to Ala residues (S6A and S25A) using the Fast Mutagenesis System Kit (TransGen Biotech) according to the manufacturer's protocol with primers listed in Supplementary Table 2, giving rise toMKK4^S6A^-pGAD and MKK5^S25A^-pGAD. All constructs were validated by sequencing and the combination of the indicated constructs were co-transformed yeast strainY2H Gold (Clontech). The yeast two-hybrid transformation and interaction assays were performed in accordance with Yeastmaker Yeast Transformation System 2 User Manual.

### Pull-down assay

Fragments encoding full-length *MPK3* and *MPK6* were amplified by PCR from wild-type cDNA and digested with BamHI and SalI. The resulting fragments were inserted into the same restriction sites of PGEX5X-1 plasmid and transformed into *Escherichia coli* (*E. coli*) BL21 (DE3). The resulting strains were grown in LB medium containing 100 μg ml^−1^ ampicillin and expression of recombinant proteins was induced by the addition of 0.4 mM isopropyl-β-D-thiogalactopyranoside at 25 °C. After 5 h, cells were collected and resuspended in PBS (135 mM NaCl, 2.7 mM KCl, 1.5 mM KH_2_PO_4_ and 8 mM K_2_HPO_4_, pH 7.4). After sonication and centrifugation, the GST fusion proteins were purified from supernatant with GST-Bind Resin (Novagen). The beads bound with GST-MPK3/MPK6 and GST were washed with PBS for GST pull-down assays. Fifty microlitre aliquots of glutathione-agarose beads were incubated with 4 μg of recombinant His-tagged ABI4 protein at 4 °C for 4 h. The beads were washed four times with 50 mM Tris-HCl, pH 7.4, 100 mM NaCl, 1 mM EDTA, 0.5% Nonidet P-40 by centrifugation at 4 °C and boiled with SDS–PAGE sample buffer for 5 min. The eluted proteins were separated on a 15% SDS–PAGE gel and subjected to immunoblot analysis using the antibody against His-tag (CW0083M, CWBIO) at a dilution of 1/1,000. The secondary antibody (goat anti-rabbit IgG, Sigma-Aldrich, A0545) conjugated to horseradish peroxidase was used at a dilution of 1/10,000 for signal detection by the enhanced chemiluminescence assay.

### Co-immunoprecipitation assay

CoIP assay was performed in protoplasts generated from wild-type and *cas-1* mutants. Briefly, the coding sequences of *14-3-3ω* were amplified by PCR amplified using primers containing a FLAG epitope and stop codon at the 3′ end and in-frame inserted into the Nco1-BamH1 sites of pSAT6-EYFP-N1 after digestion with NcoI and BamHI, resulting in FLAG-tagged 14-3-3ω construct. The coding regions of *MPK3*, *MKK4* and *MKK5* were amplified by PCR and in-frame ligated to the NcoI-XmaI sites of GFP fusion vector pSAT6-EYFP-N1with the GFP at C terminus after digestion to produce MPK3-GFP, MKK4-GFP and MKK5-GFP, respectively. The coding regions of *MPK6* were amplified were amplified by PCR using primers containing HindIII and BamHI sites and in-frame inserted into the same restriction sites of pSAT4A-cEYFP-N1 after digestion to generate MPK6-GFP. The combinations of the indicated plasmids were cotransformed into *Arabidopsis* protoplasts using PEG-calcium-mediated transformation. After overnight incubation at 23 °C, the protoplasts were harvested and lysed in 300 μl of IP buffer (10 mMHEPES–KOH, pH 7.5, 0.5% Nonidet P-40, 2 mM EDTA, 150 mM NaCl, 10% glycerol, 1 mM NaVO_3_, 5 mM NaF and 1 × protease inhibitor cocktail (Roche)) by vigorous vortexing for 1 min. The sample was centrifuged at 12,000*g* for 10 min at 4 °C to remove cellular debris. Twenty microlitres of the supernatant was collected as the input fraction. The rest of the supernatant (280 μl) was mixed with 320 μl IP buffer containing 20 μl of anti-GFP antibody coupled to protein A/G agarose beads, and incubated for 2 h at 4 °C with gentle mixing. After incubation, the beads were washed thoroughly four to five times with 1 ml of IP buffer per wash. The binging protein was eluated by boiling the beads in 30 μl of 2 × SDS–PAGE loading buffer and the presence of co-immunoprecipitated proteins was detected by immunoblotting analysis using the anti-FLAG M2 antibody(A8592, Sigma-Aldrich) covalently conjugated to horseradish peroxidase (HRP)at a 1/4,000 dilution.

### Luciferase complementation imaging assay

LCI assay was performed according to Chen *et al.*^67^. Briefly, the fragments encoding full-length *ABI4* were amplified by PCR and ligated to the KpnI-PstI site of pCAMBIA-CLuc vector after digestion to produce ABI4-CLuc. The coding regions of *MPK3* and *MPK6* were amplified by PCR and ligated to the BamHI-SalI site of pCAMBIA-NLuc vector after digestion to produce MPK3-NLuc and MPK6-NLuc. The resulting constructs were transformed into *Agrobacterium* strain GV3101. *Agrobacterium* bacteria containing indicated constructs were grown in LB medium at 30 °C overnight. After centrifugation and washing two times by infiltration buffer (10 mM MgCl_2_, 10 mM MES, pH 5.8, 100 μM acetosyringone), the bacterial suspensions were re-suspended in the same infiltration buffer, mixed with an equal volume and co-infiltrated into fully expanded leaves of the 3-week-old *Nicotianabenthamiana* using a needleless syringe. After 3 days, the leaves were sprayed with luciferin solution (100 μM luciferin, 0.1% Triton X-100) and kept in the dark for 5 min to quench the fluorescence. The LUC image was acquired by a low-light cooled CCD imaging apparatus (CHEMIPROHT 1300B/LND, 16 bits; Roper Scientific).

### BiFC assay

BiFC assays were performed essentially according to Walter *et al.*^68^ Briefly, the fragments encoding full-length *14-3-3ω* were amplified by PCR from wild-type cDNA and digested with SalI and XmaI. The resulting fragments were inserted into the same restriction sites of pSAT4A-nEYFP-N1 plasmid to produce 14-3-3ω-nYFP. The coding regions of *MPK3*, *MKK4* and *MKK5* were amplified by PCR and ligated into the EcoRI-XmaI site of pSAT4A-cEYFP-N1 after digestion to produce MPK3-cYFP, MKK4-cYFP and MKK5-cYFP. The coding regions of *MPK6* were amplified by PCR using primers containing SalI and BamHI sites and inserted into the same restriction sites of pSAT4A-cEYFP-N1 after digestion to produce MPK6-cYFP. The combinations of the indicated plasmids were co-transformed into *Arabidopsis* protoplasts using PEG-calcium-mediated transformation. YFP fluorescence was visualized 12–18 h after transformation using a confocal laser scanning microscope (LSM 510 Meta; Zeiss).

### *In vitro* phosphorylation assay

For *in vitro* phosphorylation assays, the *ABI4*cDNA was amplified and inserted into the XhoI-PstI sites of the pCold vector (Takara). Mutations of ABI4 were introduced by Fast Mutagenesis System Kit (TransGen Biotech) according to the manufacturer's instructions and verified by sequencing. Primers used for mutagenesis are listed in Supplementary Table 2. The constructs were transformed into *E. coli* BL21 (DE3). *E. coli*BL21 (DE3) cells harbouring the plasmids encoding His-tagged MPK3, His-tagged MPK6 and Flag-tagged MKK9^DD^ were the same as those used previously^60^. The BL21 (DE3) cells were collected after the application of 0.4 mM isopropyl-β-D-thiogalactopyranoside at 25 °C for 6 h and resuspended in 0.1 M Hepes–KOH, pH 7.5. After sonication and centrifugation, His-tagged wild type and mutant ABI4 proteins, His-tagged MPK3 and His-tagged MPK6 were purified through affinity chromatography by using Ni-NTA affinity agarose (Novagen) and Flag-tagged MKK9^DD^ was immunoprecipitated with anti-FLAG M2 Affinity Gel (A2220, Sigma-Aldrich) according to the manufacturer's instructions.

The *in vitro* kinase assay was performed as described previously^49^. Activated recombinant MPK3/MPK6 was generated by incubating His-tagged MPK3/MPK6 (10 μg) with recombinant MKK9^DD^ (1 μg) at 27 °C for 30 min in 50 μl of reaction buffer (20 mM HEPES–KOH, pH 7.5, 10 mM MgCl_2_, 1 mM DTT and 50 mM ATP). Then, recombinant ABI4 was phosphorylated by activated MPK3/MPK6 in the same reaction buffer containing [γ-^32^P]ATP (0.1 μCi per reaction). Addition of SDS sample buffer stopped the reactions after 30 min. After PAGE analysis (10% SDS polyacrylamide gel), phosphorylated ABI4 was detected by autoradiography. For proteins used in the immunoblot assay, phosphorylation was performed without the addition of [γ-^32^P]ATP. Uncropped images of autoradiography/immunoblots are shown in Supplementary Fig. 17.

### Mobility shift assay

Mobility shift assays to detect phosphorylated ABI4 *in vivo* were performed essentially according to Guan *et al.*^69^. Briefly, total protein was extracted from seedlings and separated on a 10% SDS–PAGE gel containing 50 μM Phos-tag reagent (NARD Institute) and 100 μM MnCl_2_. For alkaline phosphatase treatment, the protein extracts were incubated with calf intestinal alkaline phosphatase (Sigma-Aldrich) at 37 °C for 1 h and subsequently subjected to Phos-tag-SDS–PAGE. The separated proteins were then transferred to a nitrocellulose membrane and blotted with anti-GFP antibody (G1544, Sigma-Aldrich) at a dilution of 1/4,000. The secondary antibody (goat anti-rabbit IgG, Sigma-Aldrich, A0545) conjugated to HRP was used at a dilution of 1/10,000 for signal detection by the enhanced chemiluminescence assay.

### Chromatin immunoprecipitations

Seven-day-old seedlings of WT, *ABI4*^*WT*^*/abi4* and *ABI4*^*AAA*^*/abi4* grown on 1/2 MS media in the presence or absence of NF were harvested for ChIP assays following the procedure previously described^24^. Briefly, the seedlings were cross-linked with 1% formaldehyde in MC buffer (10 mM sodium phosphate, pH 7, 50 mM NaCl, 0.1 M sucrose) for 0.5 h under vacuum and washed thoroughly with MC buffer. Then, liquid nitrogen was added and the seedlings were ground to powder. Next, M1 buffer (10 mM potassium phosphate, pH 7.0, 0.1 M NaCl. 10 mM β-mercaptoethanol, 1 M 2-methyl 2,4-pentanediol) was added and the resulting slurry was filtered (Miracloth) and centrifuged (1,000*g*, for 10 min at 4 °C). The pellet obtained was washed five times with M2 buffer (M1 buffer containing 10 mM MgCl_2_, 0.5% Triton X-100), and one time with M3 buffer (M1 buffer without 2-methyl 2,4-pentanediol). The resulting crude nuclear pellet was then resuspended and sonicated in 10 mM sodium phosphate, pH 7, 0.1 M NaCl, 0.5% Sarkosyl, 10 mM EDTA, Complete Protease Inhibitor Cocktail (Roche). After centrifugation, the supernatant was mixed with an equal amount of IP buffer (50 mM Hepes–KOH, pH 7.5, 150 mM KCl, 5 mM MgCl_2_, 10 μM ZnSO_4_, 1% Triton X-100, 0.05% SDS) and precleared with Protein A/G-Agarose beads (Abmart) for 1 h at 4 °C. After centrifugation, the supernatant of chromatin complexes were incubated with anti-GFP antibody (Sigma-Aldrich) at 4 °C overnight with gentle mixing. The samples were subsequently centrifuged at 14,000*g*, for 2 min and the supernatant incubated with Protein A/G-Agarose beads (Abmart) for 1 h at 4 °C. Protein A/G-Agarose beads were subsequently pelleted, washed five times with IP buffer and vortexed for 30 s with cold glycine elution buffer (0.1 M glycine, 0.5 M NaCl, 0.05% Tween-20, pH 2.8). The supernatant was collected, neutralized with 50 μl of 1 M Tris-HCl, pH 9 and treated with RNase A at 37 °C for 15 min. After proteinase K digestion at 37 °C for 1 h, the immunoprecipitated DNA was recovered from the elutant by phenol–chloroform extraction and ethanol precipitations and analysed by quantitative real-time PCR with primers shown in Supplementary Table 2. The precipitated DNA from wild type with GFP antibody served as negative control for the PCR amplification.

### Electrophoresis mobility shift assay

The PCR fragments of ABI4^WT^ and ABI4^AAA^, which were amplified from pCold vectors containing wild-type and mutant ABI4 proteins for phosphorylation assays, were cloned into the EcoRI and XhoI sites of a maltose-binding protein fusion vector (pETMALc-H vector), resulting in pETMALc-H-ABI4^WT^ and pETMALc-H-ABI4^AAA^. Plasmids were then transformed into the *E. coli* strain BL21 (DE3). The transgenic bacteria were grown at 25 °C and induced by adding isopropyl β-D-thiogalactopyranoside (final concentration of 0.4 mM) during logarithmic growth. After cell lysis in 20 mM Tris-HCl, pH 7.4, 1 mM EDTA and 200 mM NaCl using an ultrasonic cell crusher, followed by centrifugation, the supernatant was purified by amylose resin (NEB). For labelling of the synthetic DNA oligonucleotides (5′-GACTAGAGACTGCCACATAAGAATAGTAAAGTTA-3′), the Biotin 3′ End DNA Labeling Kit (Pierce) was employed. EMSA assay was performed using Light Shift Chemiluminescent EMSA Kit (Pierce). Each binding mixtures (10 μl) containing 2 μg recombinant proteins, 1 μl of poly (dI/dC), 1 μl 10 × binding buffer, 1 μl 50% glycerol and 200 fmol of labelled probe with or without excess cold competitor double-stranded oligonucleotides were incubated at room temperature for 20 min. The DNA–protein complexes were separated on 6% native polyacrylamide gels and transferred to a positive nylon membrane. Membranes were subjected to ultraviolet light for crosslinking and probed with a streptavidin conjugated with HRP. To detect biotin-labelled DNA probes, chemiluminescence was monitored with the Light Shift Chemiluminescent EMSA Kit (Pierce). To test the impact of MAPK phosphorylation on the capacity of ABI4 to bind DNA, recombinant ABI4 was incubated with equally mixed activated MPK3 and MPK6 in the kinase reaction buffer prior to the EMSA assay. Uncropped scans of X-ray films used in figures are included in Supplementary Fig. 17.

### LUC activity assay

The reporter plasmids (*LHCB*p:LUC) and the *35S:GUS* internal control constructs were co-transformed into *Arabidopsis* protoplasts isolated from 4-week-old wild-type and the indicated genotype seedlings. After transformation, the protoplasts were incubated under light for 12–15 h. The activity of LUC and GUS was quantified using a Modulus Luminometer/Fluometer (Promega) as described previously^24^. Briefly, the protoplasts were pelleted and resuspended in Luciferase Cell Culture Lysis 5X Reagent (Promega). For GUS enzymatic assay, 10 μl of the protoplast lysate were added into 100 μl of MUG substrate mix (10 mM Tris-HCl, pH 8, 1 mM MUG, 2 mM MgCl_2_) and incubated at 37 °C for 30–180 min. The reaction was stopped by adding 0.9 ml, 0.2 M Na_2_CO_3._ For luciferase activity assay, 5 μl of the lysate was mixed with 50 μl of luciferase assay substrate (Promega). The fluorescence was measured using a GloMax 20/20 luminometer(Promega) and relative reporter gene expression was expressed as the ratio of LUC to GUS.

### Data availability

The authors declare that the data supporting the findings of this study are available within the article and its Supplementary Information files or are available from the corresponding author upon request.

## Additional information

**How to cite this article**: Guo, H. *et al.* Plastid-nucleus communication involves calcium-modulated MAPK signalling. *Nat. Commun.* 7:12173 doi: 10.1038/ncomms12173 (2016).

## Supplementary Material

Supplementary Information Supplementary Figures 1 - 17 and Supplementary Tables 1 and 2
