# Supplementary material for: Plastid-nucleus communication involves calcium-modulated MAPK signalling
Source: Nat Commun. 2016 Jul 11;7:12173. doi: 10.1038/ncomms12173 (PMC4942575; doi:10.1038/ncomms12173)
Supplement: Supplementary Information — Supplementary Figures 1 - 17 and Supplementary Tables 1 and 2 [file ncomms12173-s1.pdf]

Supplementary Figure 1

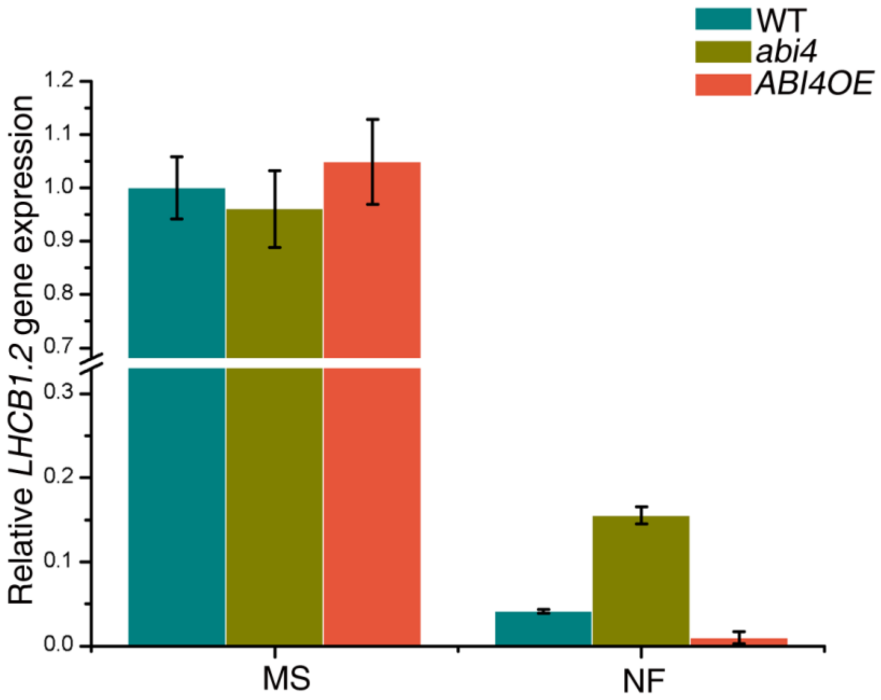

Supplementary Figure 1. Impact of NF treatment on *LHCB1.2* transcript levels in WT, *abi4* and *ABI4OE* seedlings.

The WT, *abi4* and *ABI4OE* seedlings grown on 1/2 MS medium in the presence or absence of NF were harvested after 7 days for RNA extraction and qRT-PCR analysis. Relative expression levels of *LHCB* in samples after NF treatment versus untreated samples were normalized to the expression of *UBQ10*. Values shown are means $\pm$ SD of three biological replicates.

Supplementary Figure 2

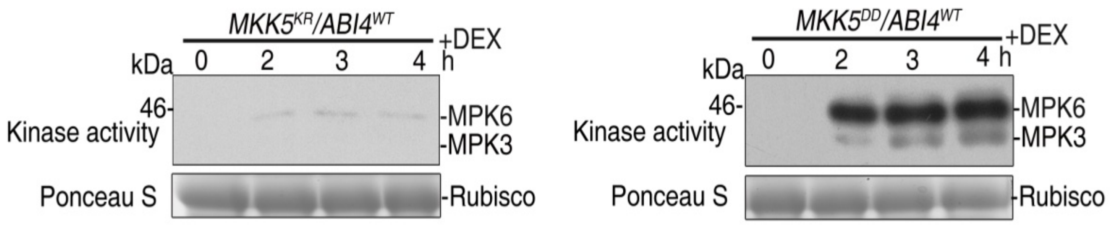

Supplementary Figure 2. DEX induces MPK6 and MPK3 activation in *MKK5^{DD}/ABI4^{WT}* but not in the *MKK5^{KR}/ABI4^{WT}* transgenic plants.

Total protein was extracted from *MKK5^{DD}/ABI4^{WT}* and *MKK5^{KR}/ABI4^{WT}* seedlings after treatment with 2 mM DEX for the indicated times and subjected to SDS-PAGE. Activated MPK6 and MPK3 were immunodetected using the phosphor-p44/42 MAPK (ERK) antibody. Equal loading was confirmed by Ponceau S staining (bottom).

Supplementary Figure 3

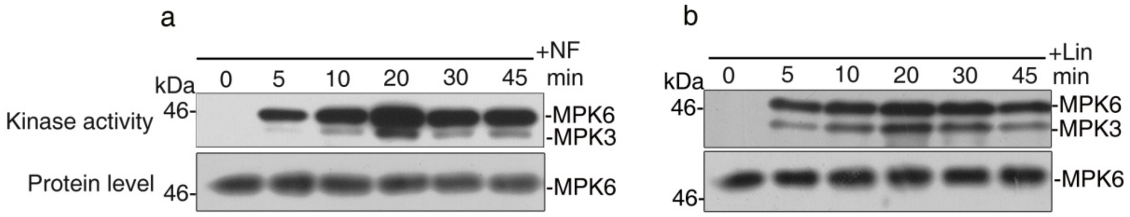

Supplementary Figure 3. NF and lincomycin induce activation of MPK6 and MPK3 *in vivo*.

(a, b) Total protein was extracted from wild-type seedlings after 5 µM NF (a) and 500 µM Lin (b) treatment at the indicated times. The kinase activity of MPK6 and MPK3 was detected by immunoblot analysis with the phosphor-p44/42 MAPK (Erk1/2) antibody. Equal loading was indicated by immunoblot analysis with anti-MPK6 antibody.

Supplementary Figure 4

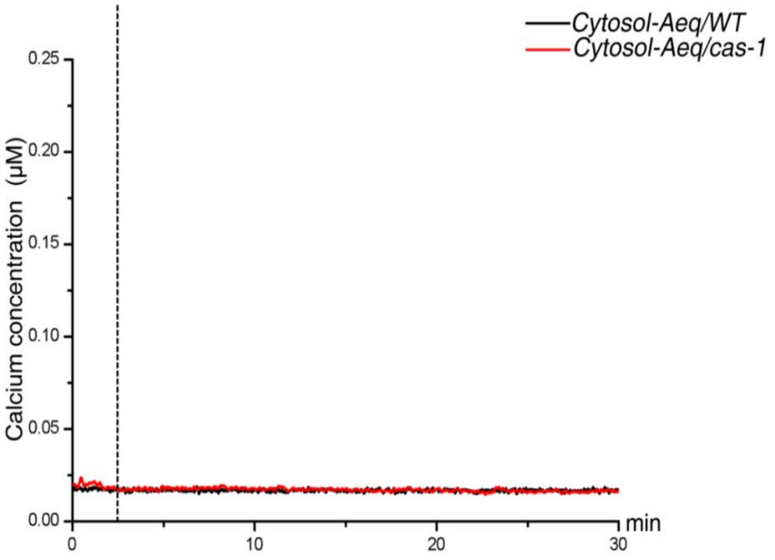

Supplementary Figure 4. Ca^{2+} dynamics in the control experiments.

6-day-old *Arabidopsis* transgenic seedlings expressing cytosolic apoaequorin in wild-type (black lines) and *cas-1* mutant (red lines) were treated with water after 3 min of counting for the base level and luminescence was recorded at intervals of 0.2sec. The vertical dashed line indicates the time at which treatment was initiated. Experiments were repeated at least five times and representative data are shown.

Supplementary Figure 5

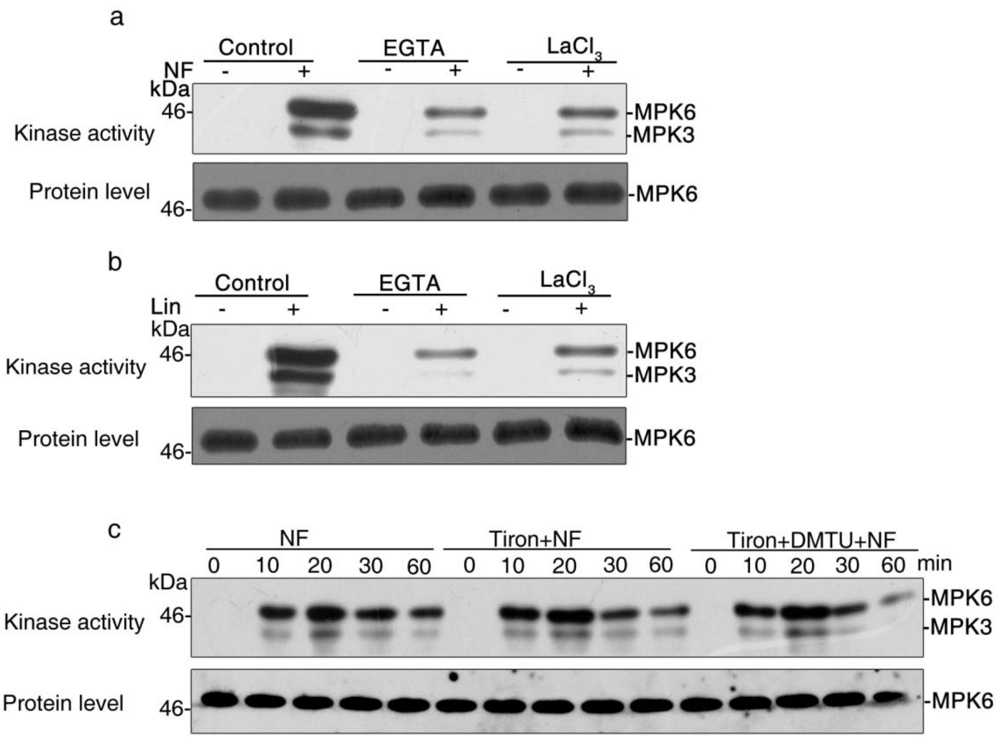

Supplementary Figure 5. Effects of $LaCl_3$, EGTA and ROS eliminators on MAPK activation.

(a, b) Effects of $LaCl_3$ and EGTA on MAPK activation during retrograde response. Protoplasts were treated with 2 mM $LaCl_3$, 10 mM EGTA or H_2O (control) for 15 min prior to elicitation with 5 μM NF (a) and 500 μM Lin (b) for 30 min. The kinase activity of MPK6 and MPK3 was detected by immunoblot analysis with the phosphor-p44/42 MAPK (Erk1/2) antibody. Equal loading was indicated by immunoblot analysis with anti-MPK6 antibody.

(c) Effects of pretreatment with ROS eliminators, Tiron and dimethylthiourea (DMTU), on the activation of MPK3/MPK6. Dark-grown 4-day-old wild-type seedlings were pretreated with 10 mM Tiron alone or in combination with 5 mM DMTU for 8 h, and then exposed to NF treatment in darkness for the time indicated. Total protein was extracted after treatments and then subjected to immunoblot analysis using the phosphor-p44/42 MAPK (Erk1/2) antibody. Equal loading was indicated by immunoblot analysis with anti-MPK6 antibody.

Supplementary Figure 6

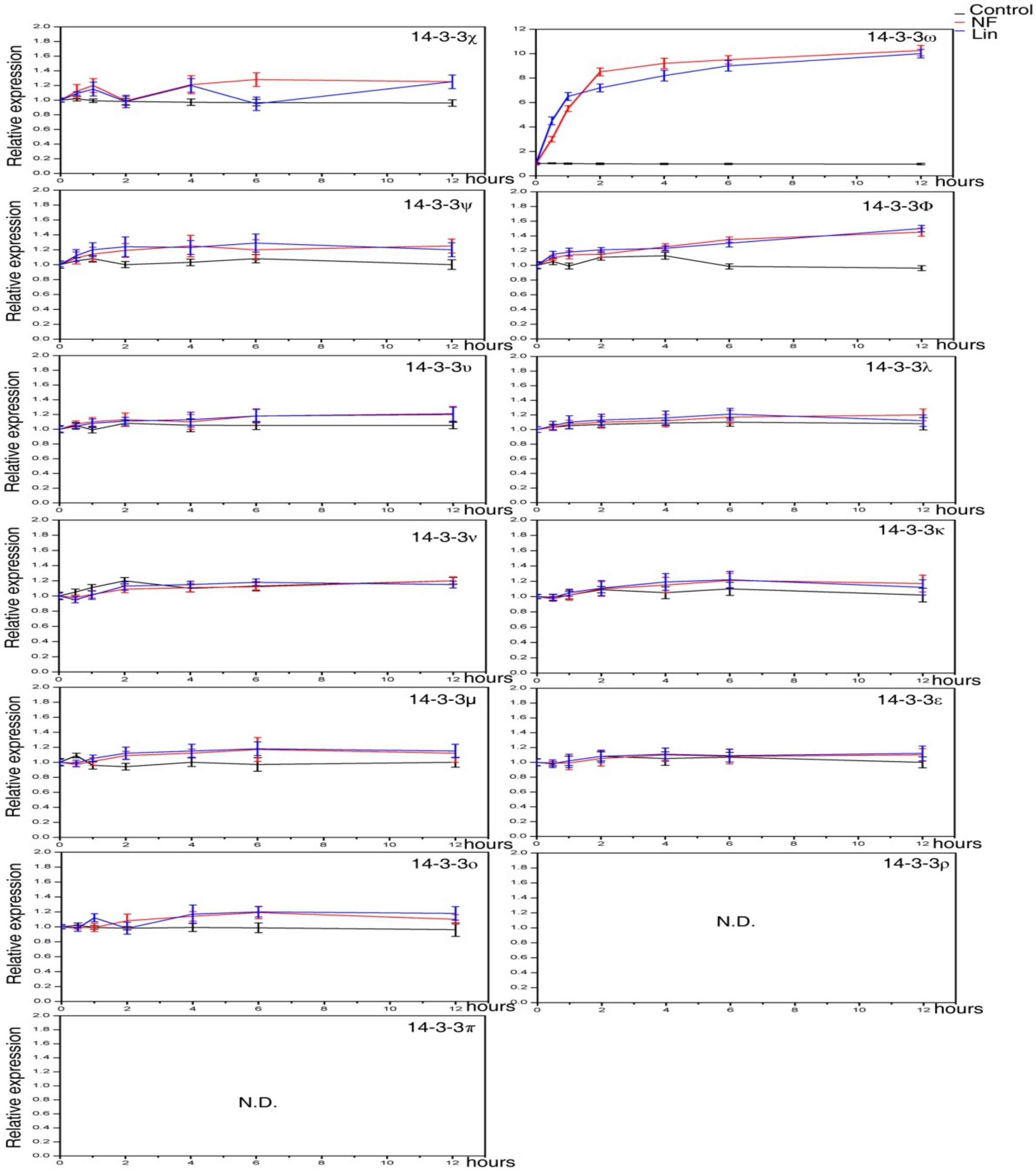

Supplementary Figure 6. Differential expression of 14-3-3 isoforms during retrograde response.

Total RNA was extracted from 7-day-old wild-type seedlings after treatments with 5 μM NF or 500 μM Lin at the indicated times. Relative transcript levels of 14-3-3 genes were determined by qRT-PCR and normalized to transcript level of control seedlings. Values shown are means ± SD of three biological replicates. N.D. , Not detected.

Supplementary Figure 7

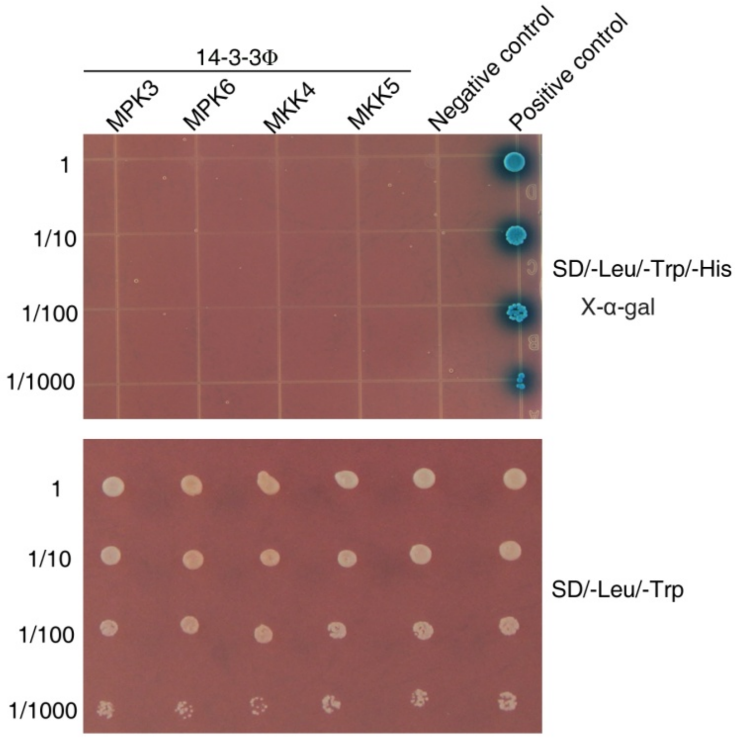

Supplementary Figure 7. Yeast two-hybrid analysis for the interaction of 14-3-3Φ with MPK3/MPK6 and MKK4/MKK5.

Fusion constructs of the 14-3-3Φ fused with the GAL4 DNA-binding domain (BD) and indicated genes fused with the GAL4 activation domain (AD) were co-transformed into Y2HGold yeast cells. Yeast strains expressing the indicated constructs were grown on synthetic dropout medium lacking Leu, Trp and His (SD-Leu-Trp–His) containing 40μg/mL X-α-Gal (5-bromo-4-chloro-3-indolyl-α-D-galactopyranoside) (upper panel) and synthetic dropout medium without Leu and Trp (SD-Leu-Trp)(lower panel).

Supplementary Figure 8

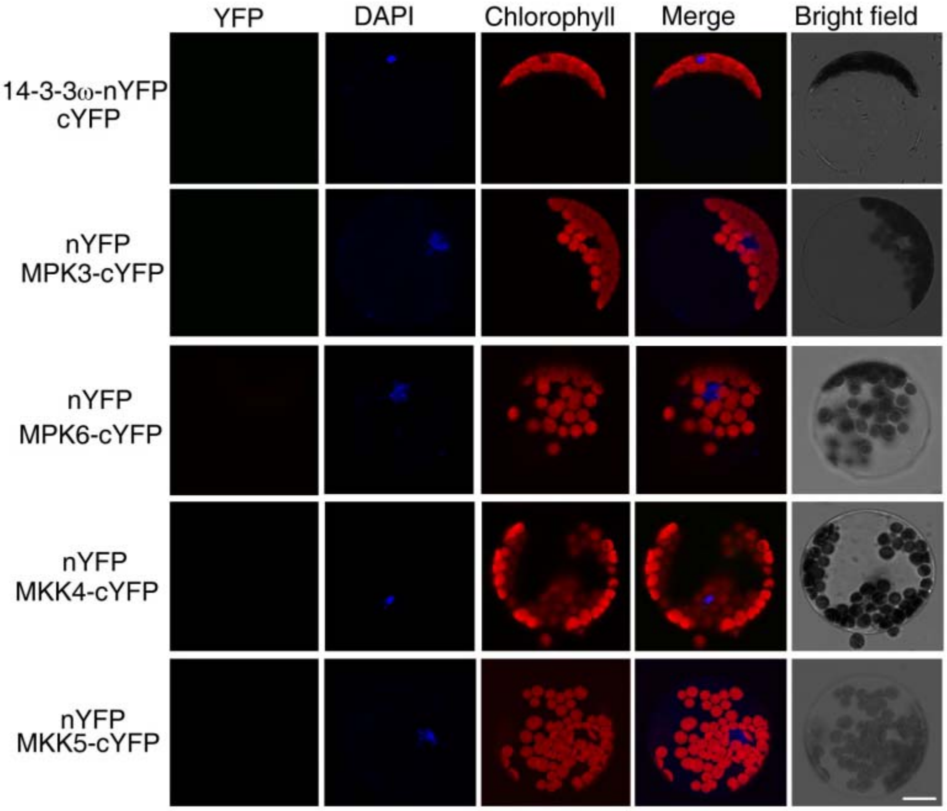

Supplementary Figure 8. The negative controls for the BiFC experiments.

No signal of YFP fluorescence was detected in *Arabidopsis* protoplasts after co-expression of 14-3-3ω-nYFP with cYFP, or nYFP with MPK3/MPK6/MKK4/MKK5-cYFP. The nuclei were indicated by DAPI staining. Scale bar, 10μm.

Supplementary Figure 9

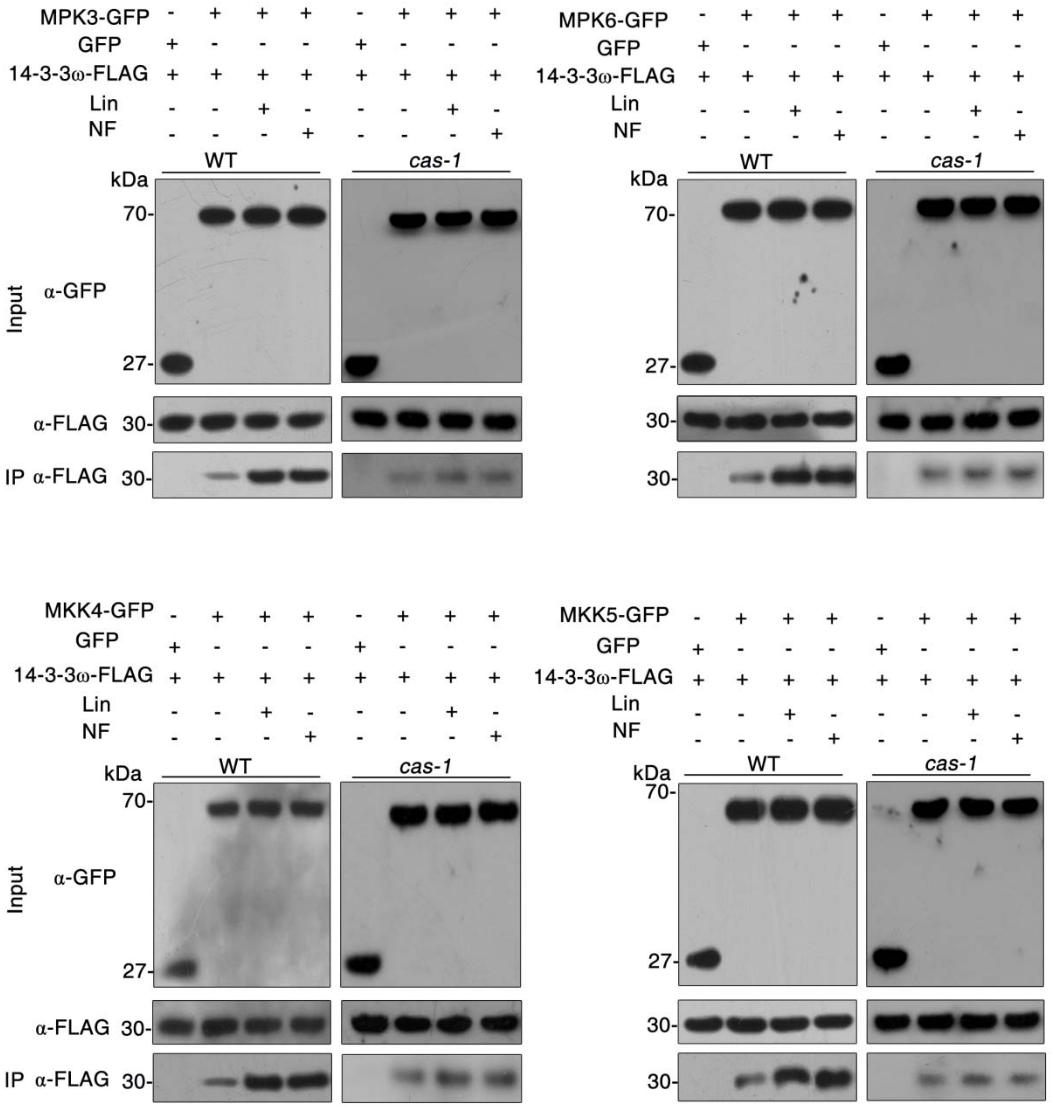

Supplementary Figure 9. Effect of Lin and NF on the association of 14-3-3ω with MPK3/MPK6 and MKK4/MKK5 in wild-type and *cas-1* mutants.

The association of 14-3-3ω with MPK3/MPK6 and MKK4/MKK5 was analyzed by coimmunoprecipitation in protoplasts. The protoplasts were pretreated with 5 μM NF or 500 μM Lin for 30min, and total protein extracts were subjected to immunoprecipitation. The immunoprecipitated proteins were analyzed by immunoblot analysis using an anti-FLAG antibody. Protoplasts were transfected with FLAG-tagged 14-3-3ω construct in combination with empty construct in each experiment to serve as a control.

Supplementary Figure 10

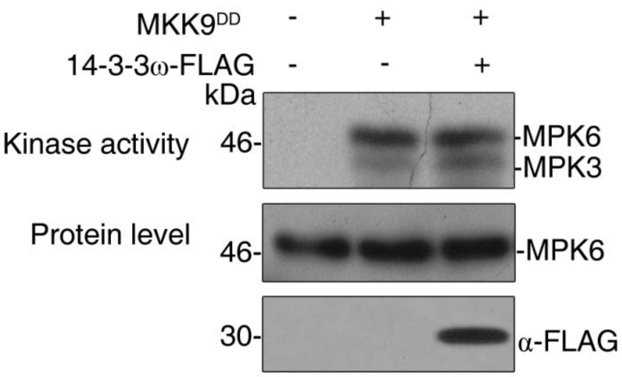

Supplementary Figure 10. Effect of 14-3-3ω on MAPK activation triggered by MKK9^{DD}.

Protoplasts isolated from 4-week-old wild-type leaves were transfected with MKK9^{DD} in the presence or absence of 14-3-3ω-FLAG constructs, and total protein extracts were subjected to immunoblot analysis with an anti-pERK antibody. Arrowheads indicate phosphorylated MPK6 and MPK3. Equal loading was confirmed by immunoblot analysis with anti-MPK6 antibody.

Supplementary Figure 11

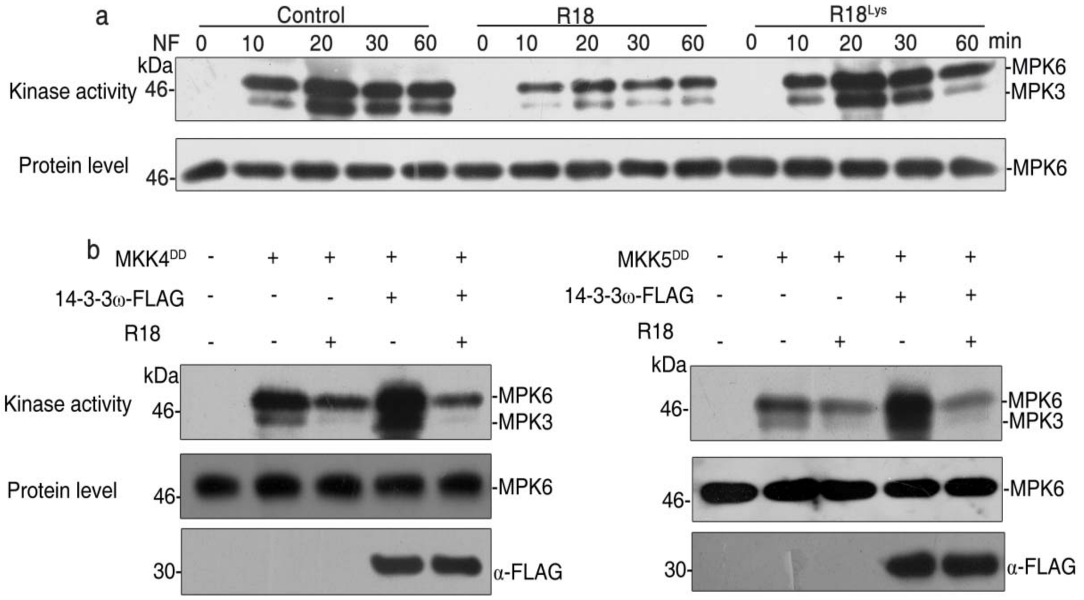

Supplementary Figure 11. Effect of R18 peptide on activation of MPK3/MPK6.

(a) Effect of R18 and $R18^{Lys}$ peptide on NF-triggered activation of MPK3/MPK6. Protoplasts were preincubated with 10 μg/mL R18 and $R18^{Lys}$ peptide for 2h and then followed by treatment with 5 μM NF for the indicated times. Total protein was extracted at the indicated times and the kinase activity of MPK6 and MPK3 was detected by immunoblot analysis with the phosphor-p44/42 MAPK (Erk1/2) antibody. Equal loading was indicated by immunoblot analysis with anti-MPK6 antibody.

(b) Effect of R18 peptide on $MKK4^{DD}$- and $MKK5^{DD}$-triggered activation of MPK3/MPK6 in the presence or absence of 14-3-3ω. Protoplasts transfected with $MKK4^{DD}$ or $MKK5^{DD}$ in the presence or absence of 14-3-3ω-FLAG constructs were preincubated with 10 μg/mL R18 peptide for 2 h and total protein extracts were subjected to immunoblot analysis with an anti-pERK antibody. Arrowheads indicate phosphorylated MPK6 and MPK3. Equal loading was verified by immunoblot analysis with anti-MPK6 antibody.

Supplementary Figure 12

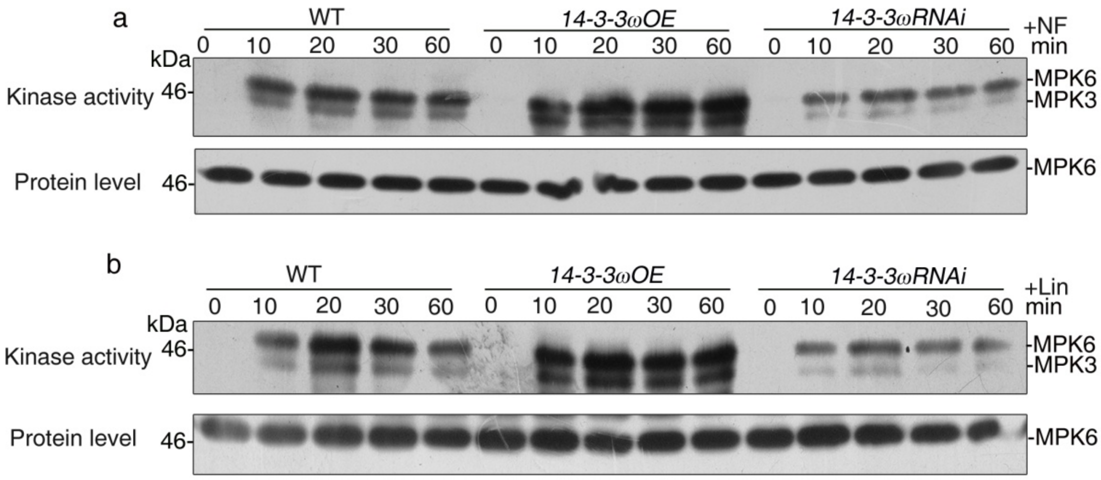

Supplementary Figure 12. MPK3 and MPK6 activation profiles in WT, *14-3-3ωOE* and *14-3-3ωRNAi* transgenic plants in response to Lin and NF treatment.

(a,b) Total protein was extracted from WT, *14-3-3ωOE* and *14-3-3ωRNAi* after treatment with 5 μM NF (a) and 500 μM Lin (b) for the indicated times and the activated MPK6 and MPK3 were detected by immunoblot analysis using phosphor-p44/42 MAPK (Erk1/2) antibody. Equal loading was verified by immunoblot analysis with anti-MPK6 antibody.

Supplementary Figure 13

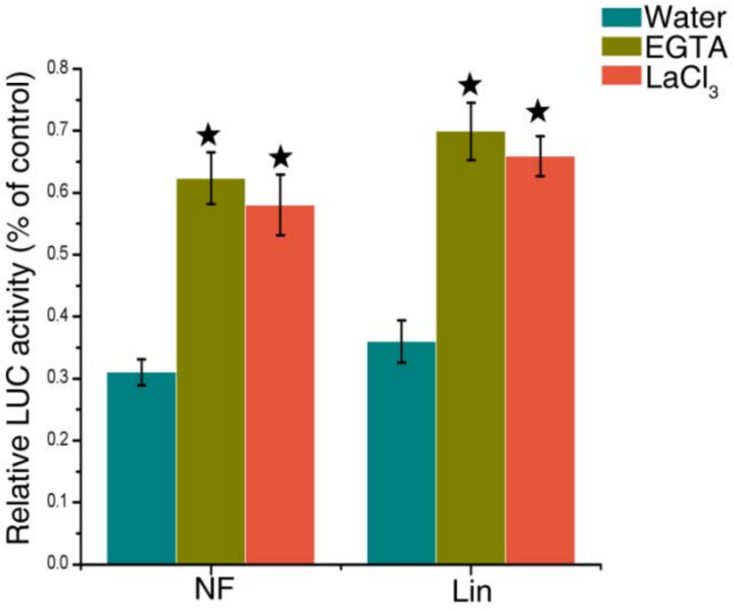

Supplementary Figure 13. Effect of preincubation of $LaCl_3$ and EGTA on LUC activity.

Protoplasts isolated from 4-week-old wild-type seedlings were cotransformed with both *LHCB*p:LUC and 35S:GUS constructs. After transformation, the protoplasts were preincubated with 2 mM $LaCl_3$, 10 mM EGTA or H_2O for 15 min and then followed by treatments with 5 µM NF or 500 µM Lin for 30 min prior to LUC assay. Relative LUC activities after treatments are the ratio of LUC to GUS (internal control) normalized to the value of control (without treatment). Data represent means $\pm$ SD from five biological replicates. Asterisks indicate significant differences from the value of preincubation with H_2O at $P < 0.05$ using Student's *t* test.

Supplementary Figure 14

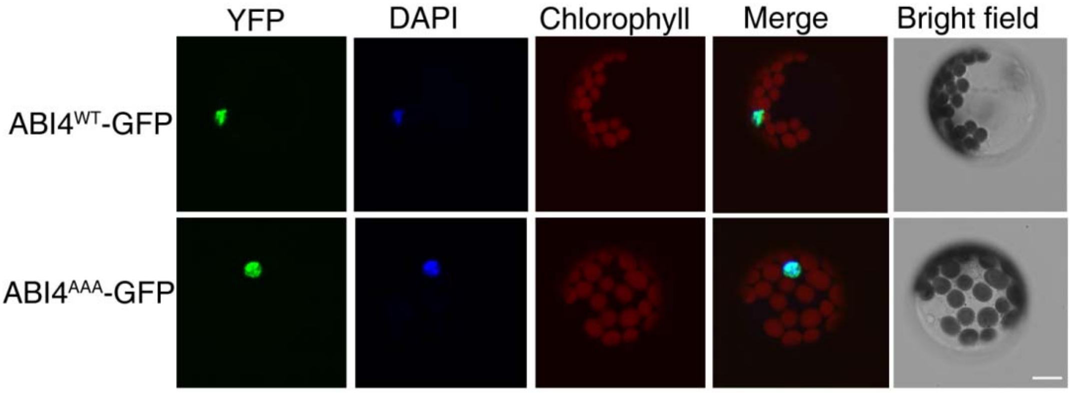

Supplementary Figure 14. Subcellular localization of ABI4^{WT}-GFP and ABI4^{AAA}-GFP.

The GFP images were obtained with a confocal microscope from *Arabidopsis* protoplasts transfected with 35S:ABI4^{WT}-GFP and 35S:ABI4^{AAA}-GFP plasmids and representative photographs are shown at the same magnification. Green indicates the GFP signal, red shows chlorophyll autofluorescence and the 4,6-diamidino-2-phenylindole (DAPI) fluorescence, shown in blue, indicates the location of the nucleus.Scale bar, 10μm.

Supplementary Figure 15

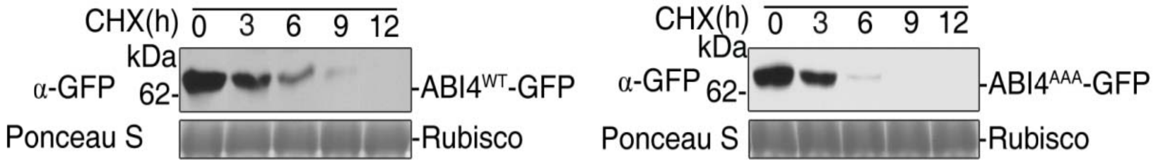

Supplementary Figure 15. The effect of mutations of phosphorylation sites on ABI4 protein stability.

Immunoblot analysis of GFP-tagged ABI4 proteins in $ABI4^{WT}$*/abi4* and $ABI4^{AAA}$*/abi4* transgenic lines. Total protein was extracted from 4-day-old seedlings pretreated with 1 mM cycloheximide (CHX) and subjected to immunoblot analysis using antibody against GFP. Equal protein loading was verified Ponceau S staining (bottom).

Supplementary Figure 16

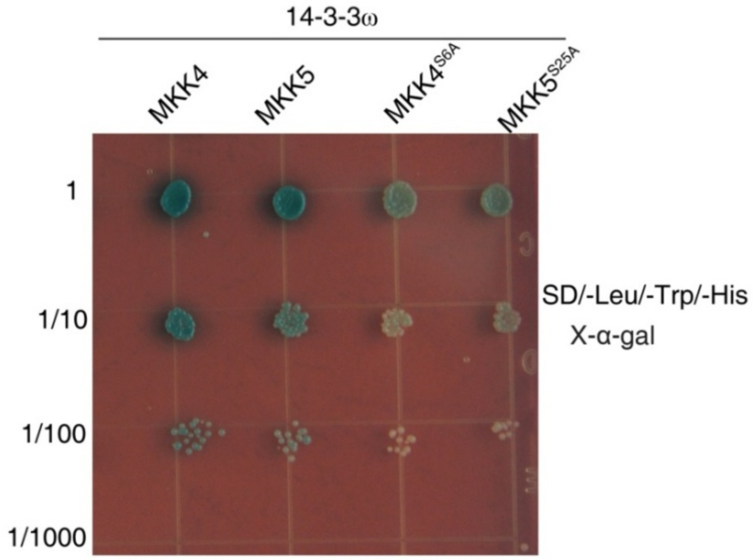

Supplementary Figure 16. Yeast two-hybrid analysis for the interaction of 14-3-3ω with MKK4/MKK5 and MKK4^{S6A}/MKK5^{S25A}

Fusion constructs of the 14-3-3ω fused with the GAL4 DNA-binding domain (BD) and indicated genes fused with the GAL4 activation domain (AD) were co-transformed into Y2HGold yeast cells. Yeast strains expressing the indicated constructs were grown on synthetic dropout medium lacking Leu, Trp and His (SD-Leu-Trp–His) containing 40 µg/mL X-α-Gal (5-bromo-4-chloro-3-indolyl-α-D-galactopyranoside).

Supplementary Figure 17

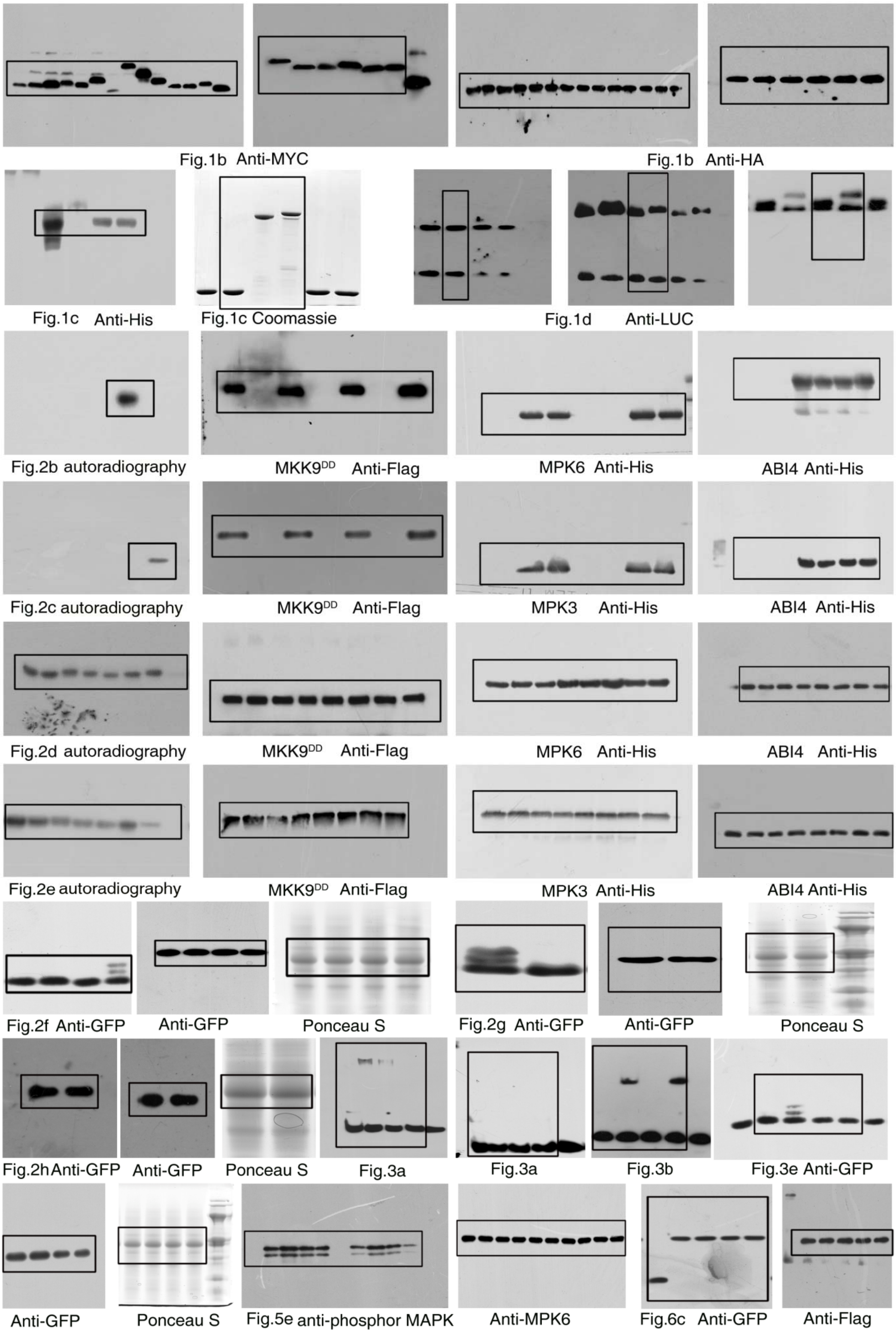

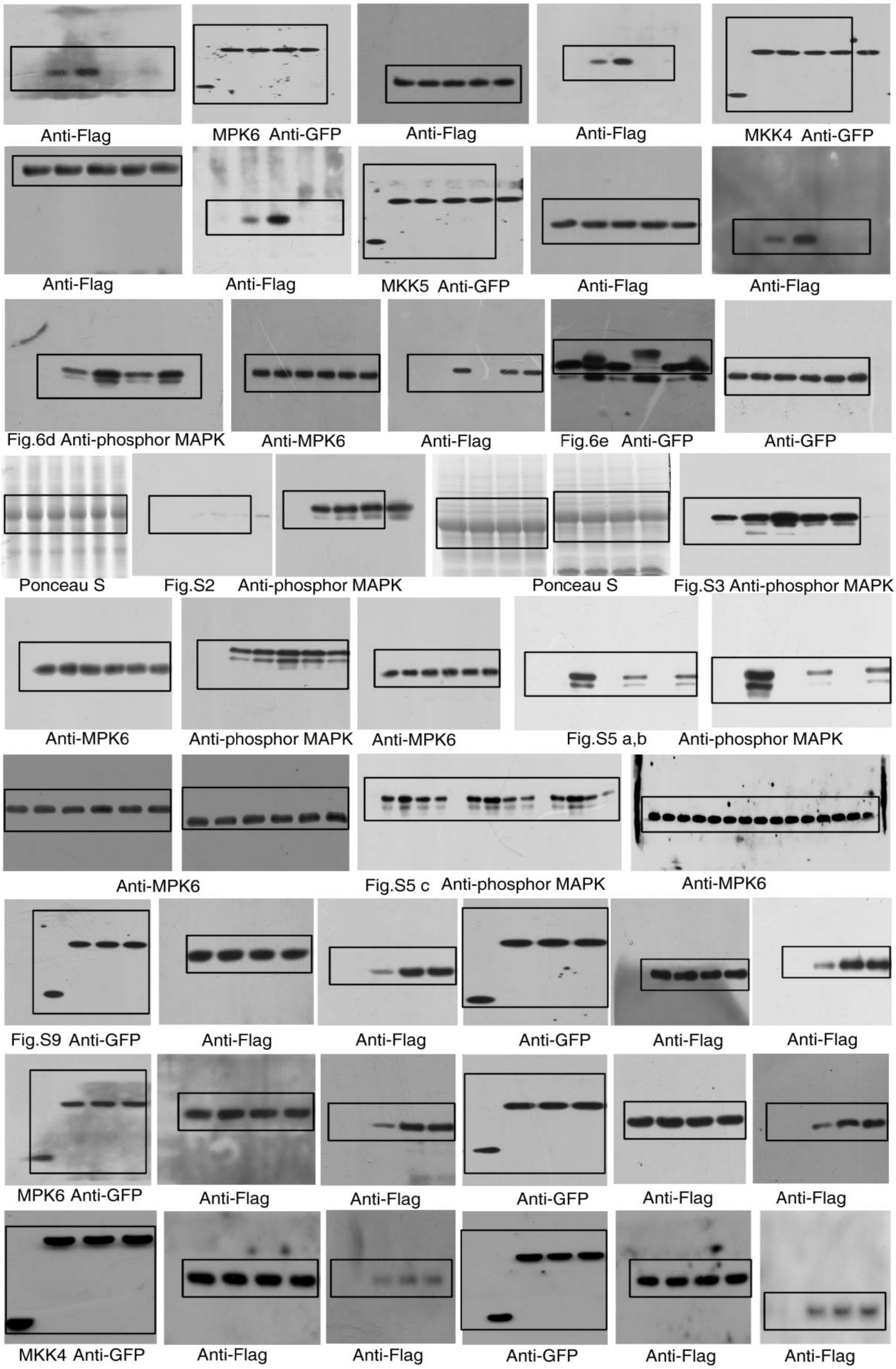
Anti-Flag
MPK6 Anti-GFP
Anti-Flag
Anti-Flag
MKK4 Anti-GFP
Anti-Flag
Anti-Flag
MKK5 Anti-GFP
Anti-Flag
Anti-Flag
Fig.6d Anti-phosphor MAPK
Anti-MPK6
Anti-Flag
Fig.6e Anti-GFP
Anti-GFP
Ponceau S
Fig.S2 Anti-phosphor MAPK
Ponceau S
Fig.S3 Anti-phosphor MAPK
Anti-MPK6
Anti-phosphor MAPK
Anti-MPK6
Fig.S5 a,b Anti-phosphor MAPK
Anti-MPK6
Fig.S5 c Anti-phosphor MAPK
Anti-MPK6
Fig.S9 Anti-GFP
Anti-Flag
Anti-Flag
Anti-GFP
Anti-Flag
Anti-Flag
MPK6 Anti-GFP
Anti-Flag
Anti-Flag
Anti-GFP
Anti-Flag
Anti-Flag
MKK4 Anti-GFP
Anti-Flag
Anti-Flag
Anti-GFP
Anti-Flag
Anti-Flag

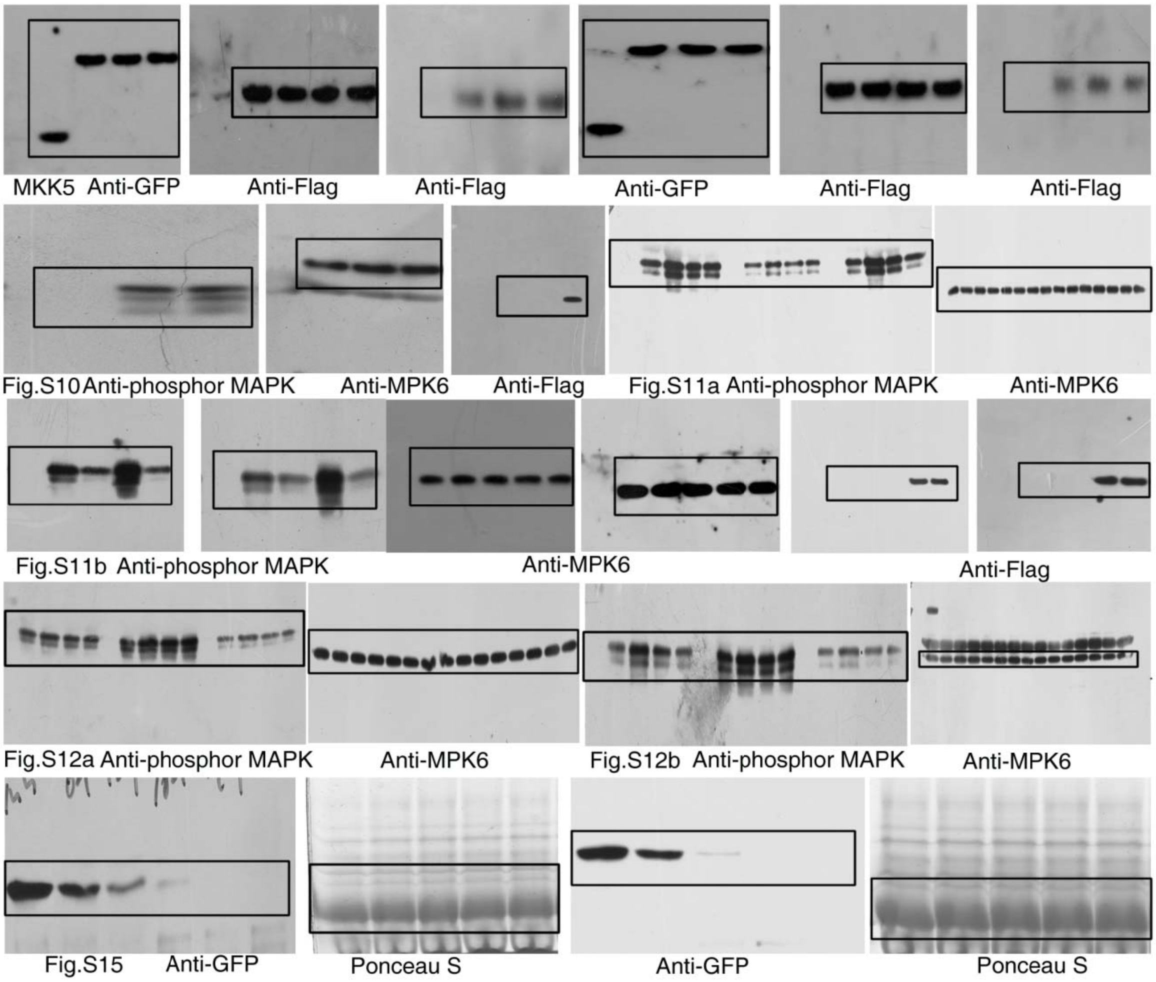

Supplementary Figure 17. Full scan data of immunoblots and autoradiography assays.

The cropped region of each figure was shown in square frame.

Supplementary Table 1. The clones identified from a yeast two-hybrid screen of an *Arabidopsis* seedling cDNA library using the N-terminal of ABI4 encoding the first 160 amino acids as bait

**Gene locus**	**Description**	**Frequency**
AT2G22360	DNAJ heat shock family protein	8
AT3G10910	DAFL1, RING/U-box superfamily protein	5
AT5G08130	BIM1,basic helix-loop-helix (bHLH) family protein	4
AT1G22640	MYB3 transcription factor	4
AT2G43790	MAP KINASE 6, MPK6	3
AT1G47380	Protein phosphatase 2C family protein	2
AT5G07060	CCCH-type zinc finger family protein with RNA-binding domain	1

Supplementary Table 2. A list of primers used in this study

Purpose	Gene	Vector	Name	Sequence (5'-3')
Yeast two hybrid assay	MPK1	pGAD	MPK1-S	5'GTACGTACCATATGATGGCGACTTTGGTTGATCC3'
			MPK1-A	5'ATGCGGATCCTCAGAGCTCAGTGTTTAAGGT3'
	MPK2		MPK2-S	5'TGGATATGCCATATGATGGCGACTCCTGTTGATCC3'
			MPK2-A	5'TAGCCCTGCAGTCAAAACTCAGAGACCTCATTGT3'
	MPK3		MPK3-S	5'GGCCCATGGGTGGCCAATACACGGATTTTC3'
			MPK3-A	5'GCCCTGCAGTGGATTGAGTGCTATGGCTTC3'
	MPK4		MPK4-S	5'GGCCCATGGAGAGTTGTTTCGGAAGCTCGG3'
			MPK4-A	5'GCCCTGCAGTGAGTCTTGAGGATTGAACTTG3'
	MPK5		MPK5-S	5'GTCACCATGGTGGCGAAGGAAATTGAATCAG3'
			MPK5-A	5'ATGCGTCGACTTAAATGCTCGGCAGAGGATT3'
	MPK6		MPK6-S	5'CAATTCCCATATGGCGGCTGATACAGAGATGACA3'
			MPK6-A	5'GGCCGTCGACGCGCCTCGCGGTAGATTAGT3'
	MPK7		MPK7-S	5'CGTAATCGCATATGATGGCGATGTTAGTTGAGCC3'
			MPK7-A	5'AGCCCTGCAGTTAGGCATTTGAGATTTCAGCTT3'
	MPK8		MPK8-S	5'GACTATGCCATATGATGGGTGGTGGTGGGAATCT3'

Yeast two hybrid assay		pGAD	MPK8-A	5'TAGCCCATGGAGAATTGTGAAGAGAAGCAACTT3'
	MPK9		MPK9-S	5'GATGCCATATGATGGATCCTCATAAAAAGGTTGC3'
			MPK9-A	5'ATGCCTGCAGTCAAGTGTGGAGAGCCGCGA3'
	MPK10		MPK10-S	5'CGACCATGGTGGAGCCAACTAACGATGCTG3'
			MPK10-A	5'GCCCTGCAGATCATTGCTGGTTTCAGGGTT3'
	MPK11		MPK11-S	5'TACGCCATGGTGTCAATAGAGAAACCATTCTTCG3'
			MPK11-A	5'ATCGGTCGACTTAAGGGTTAAACTTGACTGATTC3'
	MPK12		MPK12-S	5'ATGCCCATGGTGTCTGGAGAATCAAGCTCTG3'
			MPK12-A	5'ATGCGTCGACTCAGTGGTCAGGATTGAATTTGA3'
	MPK13		MPK13-S	5'ATGCCCATGGTGGAGAAAAGGGAAGATGGA3'
			MPK13-A	5'ATGCGTCGACATTCTTGAAGTGTAAAGACTCTCT3'
	MPK14		MPK14-S	5'ATGCATCGCATATGATGGCGATGCTAGTTGATCC3'
			MPK14-A	5'ATGCCTGCAGTTAAGCTCGGGGGAGGTAAT3'
	MPK15		MPK15-S	5'CGTAATCGCATATGATGGGTGGTGGTGGCAATC3'
			MPK15-A	5'ATGCCTGCAGAGAATTGTGTAGAGATGCAACTTT3'
	MPK16		MPK16-S	5'CGTACCATGGTGCAGCCTGATCACCGCAA3'
			MPK16-A	5'ATGCGTCGACTTAATACCAGCGACTCATTGCAG3'

Yeast two hybrid assay	MPK17	pGAD	MPK17-S	5'GCTACCATGGTGTTGGAGAAAGAGTTTTTCACG3'
			MPK17-A	5'ATGCGTCGACCTATGACACTGCAGAGGAGACAC3'
	MPK18		MPK18-S	5'GCTACCATGGTGCAACAAAATCAAGTGAAGAAG3'
			MPK18-A	5'ATGCGTCGACCTATGATGCTGCGCTGTAACTAA3'
	MPK19		MPK19-S	5'TTCTACATATGATGCAAAAAACTCAGGAGAAGAA3'
			MPK19-A	5'ATGCGGATCCCTAAGACATGCCATACCCAACAG3'
	MPK20		MPK20-S	5'GTACCCCGGGGCAGCAAGATAATCGCAAAAA3'
			MPK20-A	5'ATGCGTCGACGTACATCTTTGACATACCGTACCG3'
	MKK4	pGAD	MKK4-S	5'CGTAGAATTCATGAGACCGATTCAATCGCC3'
			MKK5-A	5'ATGCCTCGAGCTATGTGGTTGGAGAAGAAG3'
	MKK5		MKK5-S	5'CGTAGAATTCATGAAACCGATTCAATCTCC3'
			MKK5-A	5'ATGCCTCGAGCTAAGAGGCAGAAGGAAGAG3'
	ABI4	PGBK	ABI4-baitS	5'TCCACTGCATATGGACCCTTTAGCTTCCCAAC3'
			ABI4-160A	5'TATGCCTGCAGACCAAAGTTGGCTCCTCCTCCT3'
Yeast two hybrid assay	14-3-3ω		14-3-3ωPGBKS	5'CGTAGAATTCATGGCGTCTGGGCGTGAAGA3'
			14-3-3ωPGBKA	5'ATGCCTGCAGTCACTGCTGTTCCTCGGTCG3'
	14-3-3Φ		14-3-3ΦPGBKS	5'CGTACATATGATGGCGGCACCACCAGCATC3'

			14-3-3ΦPGBKA	5'ATGCCTGCAGTTAGATCTCCTTCTGTTCTT3'
Recombinant protein expression	ABI4	pCold	ABI4-S	5'CAGCCCGGGATGGACCCTTTAGCTTCCCAAC3'
			ABI4-A	5'ACGCTCGAGTTAATAGAATTCCCCCAAGATG3'
			T111A-S	5′CACGTGCTCAGCTCAACTTAGCCCCTTCGTCTCC3′
			T111A-A	5′CTAAGTTGAGCTGAGCACGTGACCCGTATAG 3′
			S114A-S	5′TCAGCTCAACTTAACCCCTTCGGCTCCTTCCTCCG3′
			S114A-A	5′CCGAAGGGGTTAAGTTGAGCTGAGCACGTGACCC3′
			S130A-S	5′CCTCCGTCTCCGCCGCTTCTGCTCCTTCCACCT 3′
			S130A-A	5′CAGAAGCGGCGGAGACGGAGGAGGAAGAGGAAG 3′
	MPK3	pGEX-5X-1	MPK3-GST-S	5'ACGCGGATCCTGAACACCGGCGGTGGCCAATA3'
			MPK3-GST-A	5'AGGCGTCGACCTAACCGTATGTTGGATTGAGT3'
	MPK6		MPK6-GST-S	5'TAGCGGATCCTGGACGGTGGTTCAGGTCAACC3'
			MPK6-GST-A	5'AGCCGTCGACCTATTGCTGATATTCTGGATTG3'
	ABI4	pETMALc-H	ABI4-MBP-S	5'GCCGAATTCGATGGACCCTTTAGCTTCCCAAC3'
			ABI4-MBP-A	5'GCCCTCGAGAAAATCCCAAATACTCCCCCA3'
Firefly luciferase	ABI4	pCAMBIA-CLuc	ABI4-S	5'GGCGGTACCATGGACCCTTTAGCTTCCCAAC3'
			ABI4-A	5'CGCTGCAGTTAATAGAATTCCCCCAAGATGGGAT3'

complementation	MPK3	pCAMBIA-NLuc	MPK3-S	5'GCGGATCCATGAACACCGGCGGTGGCCAATACACGGAT3'
			MPK3-A	5'CCGTCGACACCGTATGTTGGATTGAGTGCTATGGCTTC3'
	MPK6	pCAMBIA-NLuc	MPK6-S	5'CGCGGATCCATGGACGGTGGTTCAGGTCAACCGG3'
			MPK6-A	5'GCCGTCGACTTGCTGATATTCTGGATTGAAAGCA3'
Bimolecular fluorescence complementation (BiFC)	MPK3	pSAT4A-cEYFP	MPK3CYFPS	5'CGTAGAATTCATGAACACCGGCGGTGGCC3'
			MPK3CYFPA	5'ATGCCCCGGGACCGTATGTTGGATTGAGT3'
	MPK6		MPK6CYFPS	5'CGTAGTCGACATGGACGGTGGTTCAGGTC3'
			MPK6CYFPA	5'ATGCGGATCCATTGCTGATATTCTGGATT3'
	MKK4		MKK4CYFPS	5'CGTAGAATTCATGAGACCGATTCAATCGC3'
			MKK4CYFPA	5'ATGCCCCGGGTGTGGTTGGAGAAGAAGAC3'
	MKK5		MKK5CYFPS	5'CGTAGAATTCATGAAACCGATTCAATCTC3'
			MKK5CYFPA	5'ATGCCCCGGGAGAGGCAGAAGGAAGAGGA3'
	14-3-3ω	pSAT4A-nEYFP	14-3-3ωNYFPS	5'CGTACTGCAGATGGCGTCTGGGCGTGAAGA3'
			14-3-3ωNYFPA	5'TGCACCCGGGCTGCTGTTCCTCGGTCGGTT3'
	ABI4	pCAMBIA2300	ABI4-2300-S	5'ATCCGTCGACATGGACCCTTTAGCTTCCCAACAT3'
			ABI4-2300-A	5'ATGGCCATGGCTTCCCCCAAGATGGGATCAATAA 3'
	14-3-3ω	pSN1301	14-3-3ω1301S	5' CGTACCCGGGATGGCGTCTGGGCGTGAAGA 3'

Construction of transgenic plants			14-3-3ω1301A	5'TACGGAGCTCTCACTTGTCATCGTCATCCTTGTAATCCTTGTCATCGTCATCCTTGTAATCCTTGTCATCGTCATCCTTGTAATCCTGCTGTTCCTCGGTCGGTT 3'
	MPK6	pSN1301	MPK61301S	5'CGTAGGATCCATGGACGGTGGTTCAGGTCAA3'
			MPK61301A	5'ATGCCCCGGGCTACTTGTCATCGTCATCCTTGTAATC3'
			MPK6K92RS	5′CGAGAGCGTTGCGATTAGGAAAATTGCTA3′
			MPK6K92RA	5′CTAATCGCAACGCTCTCGTTAGTTTCAGA3′
	14-3-3ω	pFGC5941	14-3-3ωNcoI	5'CGTACCATGGCTGGACATCTGATATGCAGGATGAT3'
			14-3-3ωSwaI	5'CAGATGCATTTAAATAAAATTAAAAATTGATAAACAATCA 3'
			14-3-3ωSma1	5'ACCGTACCCGGGCTGGACATCTGATATGCAGGATGAT3'
			14-3-3ωBamH1	5'CATGCGGATCCAAAATTAAAAATTGATAAACAATCA3'
	MKK5	pTA7002	MKK5S	5'TCGATCTCGAGATGAAACCGATTCAATCTCCTTCTG3'
			MKK5A	5'ATCGCACTAGTCTAAGAGGCAGAAGGAAGAGGACG3'
			MKK5K99RS	5'CGTCCTTTCGCTCTCCGAGTGATTTACGGA3'
			MKK5K99RA	5'GAGGCTGCAGTGCAGGAAAGCGAGAGGC3'
	14-3-3ω	pSAT6-EYFP	14-3-3ωCOIPS	5'CCAACCCATGGTGGCGTCTGGGCGTGAAGAG3'

Co-immunoprecipitation Assay			14-3-3ωCOIPA	5'TTACGGGATCCTCACTTGTCATCGTCATCCTTGTAATCCTTGTCATCGTCATCCTTGTAATCCTTGTCATCGTCATCCTTGTAATCCTGCTGTTCCTCGGTCGGTT3'
	MPK3	pSAT6-EYFP	MPK3PSAT6S	5'CGTACCATGGTGAACACCGGCGGTGGCCAAT3'
			MPK3PSAT6A	5'ATGCGGATCCAACCGTATGTTGGATTGAGTG3'
	MPK6		MPK6PSAT6S	5'CGTAAAGCTTCGACGGTGGTTCAGGTCAACCGGC3'
			MPK6PSAT6A	5'ATGCGGATCCCTTGCTGATATTCTGGATTGAAAG3'
	MKK4		MKK4PSAT6S	5'CGTACCATGGTGAGACCGATTCAATCGCCT3'
			MKK4PSAT6A	5'ATGCCCCGGGTGTGGTTGGAGAAGAAGACG3'
	MKK5		MKK5PSAT6S	5'CGTACCATGGTGAAACCGATTCAATCTCCTTC3'
			MKK5PSAT6A	5'ATGCCCCGGGAGAGGCAGAAGGAAGAGGAC3'
Mutants confirmation	MPK3		SALK_100651LP	5'CATGAATAAAAGAACAGGCAAAG3'
			SALK_100651RP	5'TTGGTGTTTTTGTTGTCATGG3'
	MPK6		SALK_127507LP	5'CTCTGGCTCATCGCTTATGTC3'
			SALK_127507RP	5'ATCTATGTTGGCGTTTGCAAC3'
	ACTIN		ACTIN-2-F	5'GTCTGGATCGGAGGATCAAT3'

qPCR	ACTIN		ACTIN-2-R	5'CCTGTGAACAATCGATGGAC3'
	LHCB1.2		LHCB1.2-F	5'AATTCGGAGAAGCCGTGTGGTT3'
			LHCB1.2-R	5'TGCTTTGCGCGTGGATCAAGTT 3'
	UBQ10		UBQ10-F	5'CACACTCCACTTGGTCTTGCGT3'
			UBQ10-R	5'TGGTCTTTCCGGTGAGAGTCTTCA3'
	ABI4		ABI4-QRT-F	5'GGGCAGGAACAAGGAGGAAGTG3'
			ABI4-QRT-R	5'ACGGCGGTGGATGAGTTATTGAT3'
	14-3-3ω		14-3-3ωF	5'GACAATCTCACTCTCTGGACATC3'
			14-3-3ωR	5'CATCCGCAGCATCATCCTAATA3'
Chromatin immunoprecipitation (CHIP)	LHCB1.2		LHCB1.2CHIP-S	5'GGCTGCAATGAAAAAATCATA3'
			LHCB1.2CHIP-A	5'GTGATTGAAAATGGTTAGGTAGG3'
